# Low rates of hybridization between European wildcats and domestic cats in a human‐dominated landscape

**DOI:** 10.1002/ece3.3650

**Published:** 2018-01-27

**Authors:** Katharina Steyer, Annika Tiesmeyer, Violeta Muñoz‐Fuentes, Carsten Nowak

**Affiliations:** ^1^ Conservation Genetics Group Senckenberg Research Institute and Natural History Museum Frankfurt Gelnhausen Germany; ^2^ Institute of Ecology, Evolution and Diversity Goethe‐University Frankfurt Frankfurt am Main Germany; ^3^ European Molecular Biology Laboratory European Bioinformatics Institute Wellcome Trust Genome Campus, Hinxton Cambridge UK

**Keywords:** *Felis silvestris*, hair traps, microsatellites, mitochondrial DNA, roadkills, SNPs

## Abstract

Hybridization between wild species and their domestic congeners is considered a major threat for wildlife conservation. Genetic integrity of the European wildcat, for instance, is a concern as they are outnumbered by domestic cats by several orders of magnitude throughout its range. We genotyped 1,071 individual wildcat samples obtained from hair traps and roadkills collected across the highly fragmented forests of western Central Europe, in Germany and Luxembourg, to assess domestic cat introgression in wildcats in human‐dominated landscapes. Analyses using a panel of 75 autosomal SNPs suggested a low hybridization rate, with 3.5% of wildcat individuals being categorized as F1, F2, or backcrosses to either parental taxon. We report that results based on a set of SNPs were more consistent than on a set of 14 microsatellite markers, showed higher accuracy to detect hybrids and their class in simulation analyses, and were less affected by underlying population structure. Our results strongly suggest that very high hybridization rates previously reported for Central Europe may be partly due to inadequate choice of markers and/or sampling design. Our study documents that an adequately selected SNP panel for hybrid detection may be used as an alternative to commonly applied microsatellite markers, including studies relying on noninvasively collected samples. In addition, our finding of overall low hybridization rates in Central European wildcats provides an example of successful wildlife coexistence in human‐dominated, fragmented landscapes.

## 1 INTRODUCTION

While hybridization between closely related taxa may be a widespread and naturally occurring process, human activities may result in increased hybridization (reviewed by Brennan, Woodward, & Seehausen, 2014; Todesco, Pascual, & Owens, 2016; Wayne & Shaffer, 2016). Hybridization may constitute a source for genetic variation and adaptation (Mallet, 2005; Rieseberg, 2009), which may be considered beneficial, but it may, on the other hand, result in the breakup of genetic adaptations, genetic swamping and, ultimately, extinction (Allendorf, Leary, Spruell, & Wenburg, 2001; Rhymer & Simberloff, 1996). As a consequence, coexistence of wild and related domestic taxa that leads to the formation of hybrids is generally considered undesirable (Kopaliani, Shakarashvili, Gurielidze, Qurkhuli, & Tarkhnishvili, 2014; Muñoz‐Fuentes, Darimont, Paquet, & Leonard, 2010; Robertson, Adriaens, & Caizergues, 2015). Thus, developing methods to estimate introgression rates between wild and domestic taxa is of crucial in species conservation and monitoring to determine whether this process is occurring and to assess whether measures to revert the situation are needed. Particularly important is whether a wild species may maintain its genetic distinctiveness in the long term when outnumbered by another species with ongoing potential for crossbreeding. Thus, accurate assessment of introgression rates may have important implications for the long‐term persistence of endangered species threatened by genetic introgression from domestic congeners.

In Europe, where habitats are highly fragmented and human‐dominated, hybridization between wildcats (*Felis silvestris silvestris*; Schreber 1777) and domestic cats (*F. s. catus*) is of particular concern. First, domestic cats are virtually omnipresent throughout the wild species' range and even surpass it in numbers, presumably making encounters between the two species and introgression highly likely. In Germany, for example, the domestic cat population is estimated to be around 11.8 million individuals (ZZF e.V. 2014) and that of feral cats around 2–3 million (Tasso e.V. 2015), whereas the wildcat population is currently several orders of magnitudes lower, with approximately 1,700–5,000 individuals (Yamaguchi, Kitchener, Driscoll, & Nussberger, 2015). Moreover, declines in wildcat numbers in Europe in the late 19th century due to anthropogenic persecution and subsequent evidence for range expansions in Germany (Cocciararo et al., 2016), may have, in fact, facilitated hybridization, as severe population declines have been suggested to result in increased hybridization (see Discussion).

Putative hybrids were detected in most regions where hybridization was investigated, but the degree of introgression varied considerably. The proportion of introgressed animals was determined to be 3%–11% in Italy (Mattucci, Oliveira, & Bizzarri, 2013; Randi, 2008), 14% in the Iberian Peninsula (Oliveira, Godinho, Randi, & Alves, 2008; Oliveira, Godinho, Randi, Ferrand, & Alves, 2008), 29% in France (Say, Devillard, Léger, Pontier, & Ruette, 2012), and 21%–29% in the Jura mountains in Switzerland (21%–29%; Nussberger, Wandeler, Weber, & Keller, 2014), while in Scotland and Hungary hybrid swarms were detected (Beaumont, Barratt, & Gottelli, 2001; Pierpaoli, Birò, & Herrmann, 2003). Differing hybridization rates might be explained by varying environmental conditions, relative numbers of wild and domestic cats, or population histories (Pierpaoli et al., 2003). However, in western Central Europe, several studies came to very different conclusions regarding the degree of domestic cat introgression in the wildcat population. While Pierpaoli et al., (2003), Eckert, Suchentrunk, Markov, & Hartl, (2010), and Cocciararo et al., (2016) found no or few evidence for hybridization in Germany and adjacent areas, Hertwig, Schweizer, & Stepanow, (2009) reported low introgression in cats sampled in central Germany but concluded that almost half of the analyzed individuals from western Germany were introgressed. These findings have important consequences for current regional and large‐scale conservation programs. For example, in the frame of ongoing activities aiming at reconnecting wildcat habitats to create a nationwide biotope network (Vogel & Mölich, 2013), determining whether there are specific regions with high hybrid presence would be of crucial importance.

Despite the relevance of obtaining accurate hybridization rates for wildcat conservation, making precise comparisons across the above‐listed studies is, however, somewhat difficult, as hybridization estimates are based on different sets of microsatellite loci (7–35 loci depending on the study) or, more recently, on genomewide SNPs (Nussberger, Wandeler, Weber et al., 2014). Moreover, the number of individuals analyzed, the type of analyses conducted, and the specific methods used to calculate hybridization rates varied significantly from study to study. In order to try to understand the reasons for previous divergence in hybridization estimates, in particular those reported for western Central Europe, we aimed at estimating the degree of introgression in wildcats collected throughout this region by increasing sample coverage and using different approaches concerning sampling strategies, marker systems, and analytical methods. We used microsatellite markers as well as recently developed genomewide SNP markers (Nussberger, Greminger, Grossen, Keller, & Wandeler, 2013). Samples were obtained by opportunistically collecting tissue from roadkills (i.e., cats found dead from traffic accidents) as well as from the systematic use of hair traps (lure sticks impregnated with valerian, an olfactory cat attractant; see Material and Methods). Most studies studying hybridization in wildcats have relied on carcasses from roadkills (Eckert et al., 2010; *n* = 266; Hertwig et al., 2009; *n* = 149; e.g., Pierpaoli et al., 2003; *n* = 336; Say et al., 2012; *n* = 465), and only a few studies are based on additional noninvasively collected samples (Nussberger, Wandeler, Weber et al., 2014; Cocciararo et al., 2016). These sampling strategies may suffer from different biases. Roadkill samples are collected opportunistically, and it is possible that hybrids resembling domestic cats may be underrepresented in the samples, as collectors may primarily aim at sampling wildcats. In contrast, hair traps are usually placed in assumed wildcat habitat (mainly closed forest regions in Central Europe), potentially resulting in an underestimation of hybrids. In addition, we attempted to cover the entire range of the species in the study region (Germany and Luxembourg) and assess population substructure.

Specifically, we aimed at answering the following questions: (i) What is the rate of hybridization in wildcats in a highly fragmented, human‐dominated landscape such as Central Europe? (ii) What is the primary direction of hybridization and introgression between wild and domestic cats? and (iii) To what degree do different approaches (i.e., marker systems—SNPs vs. microsatellites, sampling regimes—roadkill vs. hair traps, and unawareness of population structure) may affect the conclusions regarding introgression rate estimates and, if such is the case, which of these factors can explain diverging estimates of hybridization rates in Central European wildcats?

## 2 MATERIAL AND METHODS

### 2.1 Samples

Samples included tissue, blood, and hair (Table S1). Tissue samples were opportunistically collected between 1995 and 2014 from wild and domestic cats found dead in traffic accidents in Germany and Luxembourg, while blood samples were obtained from captured individuals (e.g., as by‐products of various published and unpublished telemetry studies) and from domestic cats in a veterinary clinic (*n *=* *27) in Hofheim am Taunus, Germany. Tissue samples were stored in 96% ethanol, and blood samples were preserved in EDTA. Hair samples were collected opportunistically with lure sticks (Steyer, Simon, Kraus, Haase, & Nowak, 2013), from 2007 to 2013, mostly in forest habitat. Hair samples were kept dry in paper envelopes inside plastic bags with silica gel until DNA extraction. Our final dataset comprised tissue or blood samples corresponding to 536 individuals (roadkills, *n = *489; captured wildcats, *n = *20; domestic cats from a veterinary clinic, *n = *27) and hair samples (*n = *1,022 processed, *n *=* *535 kept for analysis after applying quality filters for noninvasive samples and individualization, as described below).

### 2.2 Laboratory procedures

DNA from roadkill samples was extracted using the Qiagen Blood and Tissue Kit (Hilden, Germany) following the manufacturer's instructions. DNA from hair was obtained in a dedicated laboratory for samples with low DNA quantity using the Qiagen Investigator Kit with an additional 5‐min incubation step at the final elution step.

We obtained a 110‐bp sequence corresponding to the control region of the mitochondrial DNA (mtDNA) using primers LF4 (Eckert et al., 2010) and H16498 (Kocher, Thomas, & Meyer, 1989), as described in Steyer et al., (2013). This fragment has proven to provide considerable resolution concerning the differentiation of wildcat and domestic cat in an earlier study, showing seven fixed nucleotide differences between wildcat and domestic cat (Cocciararo et al., 2016). The obtained fragments were compared with reference sequences deposited in GenBank. Five haplotypes were observed for the first time in this study (SNG‐HP‐FS18, ‐FS49, ‐FS59, ‐FS60, ‐FS61, accession numbers KX161418‐KX161423), while 26 others had been observed previously (see Table S2 for accession numbers).

Samples which yielded a single cat haplotype were then genotyped for 14 microsatellites using the polymerase chain reaction (PCR) in four multiplexes: (i) FCA8, FCA171, FCA571, and FCA124; (ii) FCA149, FCA170, FCA88, and FCA275; (iii) FCA364, FCA132, and FCA576; and (iv) FCA232, FCA347, FCA567 (Menotti‐Raymond, David, & Lyons, 1999), and a Zn finger sex marker (Pilgrim, McKelvey, Riddle, & Schwartz, 2005), following PCR protocols in Steyer et al., (2013). Fragments were size‐separated on an ABI 3730 DNA Analyzer (Applied Biosystems), and allele sizes determined using LIZ500 as a size standard in GeneMarker 2.2 (SoftGenetics). For most blood (83%) and tissue samples (93%), a minimum of two genotype replicates were obtained, and a minimum of three for all hair samples, which enabled us to calculate allelic dropout (ADO) and false allele (FA) rates using a custom R script (R Core Team 2014) based on the consensus and error estimation rules described in Gimlet (Valière, 2002), accepting a heterozygote locus if it was found at least once. Samples from both datasets with data for eleven or more loci (roadkill dataset *n *=* *536, hair dataset *n *=* *1,022) were kept for further analyses and, in the case of the hair samples, consensus genotype construction. Individual assignment was conducted using an R custom script. Individuals' consensus genotypes with >75% amplification success and <20% ADO rates across all loci were kept for further analyses. A 100% amplification success indicates that all genotype replicates which were assigned to the same individual showed allele information at all 14 loci, whereas a dropout rate of zero indicates identical alleles across all genotype replicates belonging to one individual. Repeated detections of the same genotype were assumed to be the same individual and were removed from further analyses.

The roadkill samples, as well as all available hair samples that could not be assigned using NewHybrids and provided sufficient material for an additional analysis (*n* = 53), were analyzed with a 96 genomewide SNP panel (SNPtype genotyping assays, Fluidigm, San Francisco, CA, USA) on a 96.96 Dynamic Array Chip for Genotyping (Fluidigm), as developed by Nussberger, Wandeler, & Camenisch, (2014), which allows to simultaneously genotype 96 SNP loci in 96 samples. The SNP set contained 75 markers previously selected for their diagnostic value to differentiate wild and domestic cats, 15 markers adequate for wildcat individual identification, and four maternally and two paternally inherited markers (Nussberger, Wandeler, & Camenisch, 2014). All 96 SNP loci were pre‐amplified in specific target amplification (STA) reactions using 1.25 μl of template DNA, following the manufacturer's instructions. On each plate, a minimum of two nontemplate controls and two nontemplate STA reactions were included to check for potential contamination. Genotyping was performed using the Fluidigm SNP Genotyping Analysis Software v.3.1.2. We excluded samples and SNP markers with <10% data during the scoring phase, and afterwards SNP markers that were not callable in 30% or more of the samples, as well as genotypes showing <70% amplified loci.

### 2.3 Data analyses

Structure 2.3 (Pritchard, Stephens, & Donnelly, 2000) was used to potentially identify wildcats and domestic cats as well as admixed individuals based on multilocus genotypes. Structure identifies the most likely number of populations (*K*) using a Bayesian clustering method and probabilistically assigns individuals to populations without using sampling location information. We performed ten runs for each value of *K* (from 1 to 10) using the admixture model with correlated allele frequencies (Falush, Stephens, & Pritchard, 2003) with 30,000 steps for burn‐in and 500,000 steps for run length. We used Structure Harvester (Earl & vonHoldt, 2011) to visualize the results and to obtain input files for Clumpp 1.1.2 (Jakobsson & Rosenberg, 2007). CLUMPP was used to align the cluster membership coefficients from the 10 replicate analyses for each *K*. At *K *=* *2, a cluster composed of wildcats and another one comprising domestic cats were identified, while at *K *=* *3, two wildcats clusters, one with individuals predominantly sampled in western Germany and one with individuals mostly sampled in central Germany, as well as a domestic cat cluster, were found. Individuals were considered assigned to one cluster (wildcat or domestic cat) if the assignment value (*q* ^(*i*)^) was ≥.75 to minimize the number of unassigned individuals. Individuals with assignment of *q* ^(*i*)^ < .75 to any cluster and *q* ^(*i*)^ ≥ .19 to the domestic cat cluster were classified as admixed (potential domestic and wildcat hybrid). Individuals which could not be assigned to any of the groups (e.g., *q* ^(*i*)^ < .75 to any cluster and *q* ^(*i*)^ < .19 to the domestic cat cluster) were considered to be potentially admixed between the eastern and western wildcat clusters. The assignment threshold was set lower than *q* ^(*i*)^ ≥ .8 because presumably admixed samples of wildcat and domestic cat were analyzed in the downstream NewHybrids analysis, while samples which were potentially admixed between the eastern and western wildcat cluster were excluded. Comparison of *q* ^(*i*)^ thresholds *q* ^(*i*)^ ≥ .75 and *q* ^(*i*)^ ≥ .8 affected only assignment of 19 individuals (see raw data spreadsheet Dryad https://doi.org/10.5061/dryad.fp954). We used Arlequin 3.5 (Excoffier & Lischer, 2010) to obtain microsatellite allele frequencies and calculate genetic diversity estimates on samples assigned to the wildcat or the domestic cat clusters as detected using Structure.

The software NewHybrids (Anderson & Thompson, 2002) was used to identify pure individuals and different hybrid classes. We estimated posterior probabilities to six genotype classes, including the two parental species, F1, F2, and backcrosses to either parental. The program was run for at least 100,000 sweeps, with no a priori information about the origin of the individuals. As recommended in the manual, the sensitivity of the results to different prior combinations was examined.

To investigate the direction of hybridization, migration rates were calculated using BayesAss (Wilson & Rannala 2003). In addition, mtDNA sequences obtained in this study were aligned to 29 previously published sequences (Table S2; Nussberger, Weber, Hefti‐Gautschi, & Lueps, 2007; Cocciararo et al., 2016) using the clustalW algorithm (Thompson, Higgins, & Gibson, 1994) in geneious 7.1.7 (Biomatters). Haplotypes were assigned to wildcat or domestic cat when they were found in a minimum of 10 individuals, the majority of which assigned to either wildcat or domestic cat based on the individuals' genotypes, according to NewHybrids results based on the SNP dataset; if a haplotype was found in <10 individuals, the subspecies was not specified. A statistical parsimony network was obtained using TCS 1.21 (Clement, Posada, & Crandall, 2000), with a connection limit of four and treating gaps as a fifth character state.

### 2.4 Simulations

In order to investigate the power of our markers to detect wildcat and domestic cat hybrid classes, we generated hybrid genotypes using Hybridlab (Nielsen, Bach, & Kotlicki, 2006). For this purpose, we selected complete empirical microsatellite genotypes (no missing loci) from the roadkill dataset that assigned to either parental subspecies based on all available data (mtDNA, microsatellites, and SNPs), as determined by Structure (*q* ^(*i*)^ ≥ .75 assignment probability to one of the two wildcat clusters or the domestic cat cluster, *K *=* *3) and NewHybrids (*q* ^(*i*)^ ≥ .85 assignment to one of the parental species). We identified 154 genotypes among the individuals that assigned to the western cluster and 130 genotypes among those that assigned to the central cluster. We then used each wildcat group of genotypes separately to generate different hybrid classes with 77 empirical genotypes selected from our sample of domestic cats. We generated 200 individuals of each of the following categories: parentals, F1s, F2s, and first and second backcrosses to either parental subspecies. For SNP data simulations, we used the same individuals as selected above, but SNP genotypes from the two wildcat populations (western and central) were merged, as no structure was detected in wildcats using the SNP markers. Then, simulated hybrid classes were generated as described above.

We ran Structure as indicated earlier for each of the three datasets (western wildcat microsatellites, central wildcat microsatellites, and SNP dataset), but only for *K *=* *2 as we wanted to test the sensitivity of Structure to assign individuals to one of two clusters (wildcats or domestic cats). We graphically displayed the individual assignment probabilities for Structure *q* ^(*i*)^ ≥ .75 as boxplots using R version 2.15.3. We then ran NewHybrids on the 200 simulated genotypes of each parental genotype and 50 randomly selected genotypes of each of the four hybrid classes and represented them as boxplots as described above, using a threshold probability of *q* ^(*i*)^ *≤ *.85.

## 3 RESULTS

The final dataset included 1,071 individuals which were successfully genotyped using 14 microsatellite markers and 589 individuals using 60 SNP markers. The roadkill dataset analyzed with microsatellites consisted of 536 individuals, with a mean amplification success of 95% and a mean ADO rate of 2%; in the case of the SNPs, the mean amplification success was also 95%. The hair trap dataset analyzed with microsatellites comprised 535 individuals, and the success rates resulted in a mean amplification success of 96% and a mean ADO rate of 10%.

### 3.1 Genetic population structure

**Figure 1:**
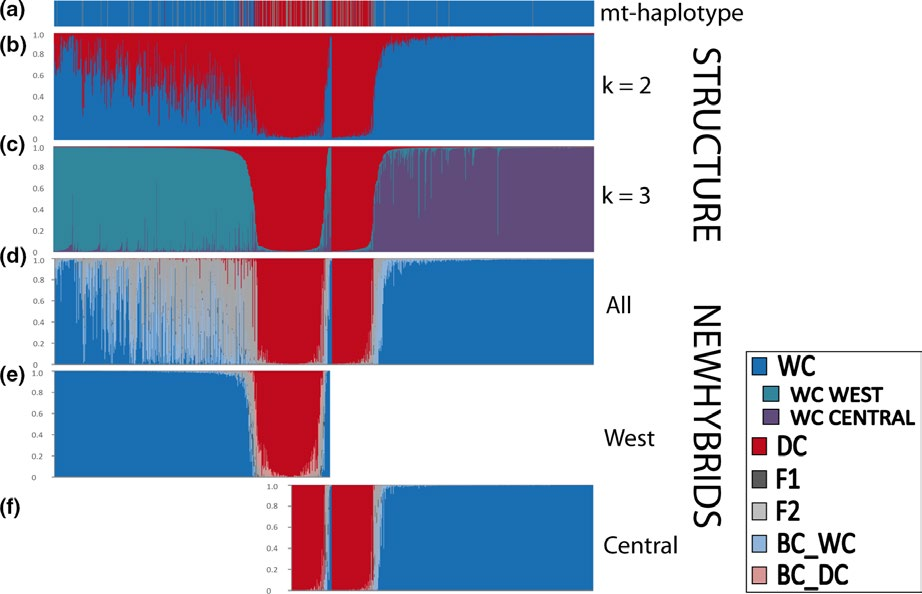
Assignment of 1,071 individuals based on control region mtDNA (a) and microsatellite (b–f) data, using Structure (b, c) and NewHybrids (d, e, f) (for haplotype assignment to wildcat or domestic cat, see text). Individuals identified as belonging to the western or the central cluster by structure were analyzed together (d) and separately using NewHybrids (e and f). Wildcats are sorted from west to east; domestic cats and individuals belonging to the western and central groups are placed in the center

Structure analyses of all samples genotyped with microsatellite markers (*n *=* *536 roadkill; *n *=* *535 hair traps) indicated that the greatest likelihood was for *K *=* *3 (following Evanno, Regnaut, & Goudet, 2005), with 379 wildcats sampled in Luxembourg and western Germany assigned to one group, 414 wildcats sampled in central Germany to another, and 211 widespread distributed individuals to the remaining one, comprising individuals sampled in the wild and including also the 27 domestic cat samples obtained from a veterinary clinic (Figure 1c). In addition, 32 individuals were presumably wildcats (*q* ^(*i*)^ < .25 to the domestic cat cluster), but they could not be clearly assigned to the western or the central cluster (*q* ^(*i*)^ *≤ *.75 to either), and 35 individuals were presumably admixed wildcat and domestic cat as they had similar probability of belonging to a wildcat or the domestic cat cluster.

For the roadkill dataset and the samples from the veterinary clinic genotyped using SNPs, STRUCTURE analyses indicated that the greatest likelihood was for two populations (*K* = 2 Evanno et al., 2005): 406 wildcat individuals assigned to one group, and 124 individuals to another, which included all domestic cats from the veterinary clinic (*n = *27). The individuals above which assigned to either a wildcat or a domestic cat cluster (*q* ^(*i*)^ **≤* *.75) based on microsatellite data, also assigned to a wildcat or domestic cat cluster, respectively, based on SNP data. In addition, we found six individuals presumably admixed between wildcat and domestic cat (*q* ^(*i*)^ **≤* *.75 to any of the two clusters).

*F*_ST_ values between the three clusters identified by structure (west, central, domestic cats) were significant (*p* < .001 in all cases; Table S3). Differentiation between domestic cats and any of the two wildcat groups was greatest (for hair traps and roadkills *F*_ST_ = 0.091–0.170 with microsatellites, and 0.783 and 0.803 with SNPs, respectively; see Table S3). Wildcats from central Germany differed more strongly from domestic cats than wildcats from the western cluster. Differentiation between the two wildcat populations was *F*_ST_ = 0.087 (hair traps) or *F*_ST_ = 0.084 (roadkills) with microsatellites, and 0.066 (roadkills dataset) with SNPs. Allele frequency tables for the microsatellite and the SNP datasets are provided (Tables S4 and S5).

### 3.2 Hybridization analyses

NewHybrids analyses were conducted in two ways for microsatellite data: (i) all samples together and (ii) samples analyzed separately according to whether the samples belonged to the western or the central wildcat cluster as detected by Structure. In the latter analyses, individuals in the domestic cat cluster and individuals that were potentially admixed between wildcats and domestic cats were included in either the “western” or “central” analysis according to their geographical sampling location (see Figure S1 for a map). Individuals collected in the area where the two wildcat groups overlapped geographically (*n *=* *80) were analyzed in both the western and central datasets. Individuals which were potentially admixed between the eastern and western wildcat cluster (9 individuals from the roadkill dataset, 19 individuals from the hair dataset) were excluded. We used an assignment threshold of *q* ^(*i*)^ ≥ .85 and tested the sensitivity of the results to different prior combinations. For the SNP data, all individuals were run together in NewHybrids, as the greatest likelihood was for *K *=* *2 as based on structure. The microsatellite‐based NewHybrids analyses were not consistent when prior combinations or sample groupings were changed and the likelihood values were low (see raw data spreadsheet Dryad https://doi.org/10.5061/dryad.fp954).

In conclusion, the microsatellite‐based NewHybrids analyses for the western and central groups run separately, based on Uniform–Uniform and Uniform–Jeffreys priors and assignments with *q* ^(*i*)^ ≥ .85 (89% in the western analysis, 95% in the central analysis) resulted in 80% wildcats and 20% domestic cats in the western group (Figure 1e), and in 75% wildcats, 25% domestic cats, one F2 and one backcross to wildcat in the central group (Figure 1f).

**Figure 2:**
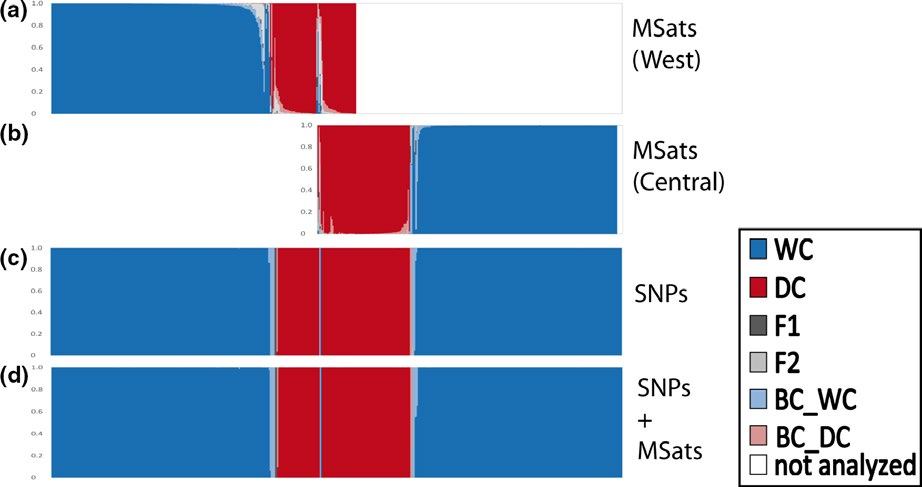
Assignment of 536 roadkill samples analyzed using NewHybrids based on microsatellite (a, b), SNP (c) or microsatellite and SNP data combined (d). Because microsatellite data indicated two wildcat clusters, these individuals were analyzed separately in the microsatellite‐based NewHybrids analysis (a and b). Individuals that were not clearly assigned to either of the two wildcat clusters as identified by Structure (*n = *19, see text for details) were not analyzed using NewHybrids. Wildcats are sorted from west to east; domestic cats and individuals belonging to the western and central groups are placed centrally

SNP‐based NewHybrids analyses were consistent across all prior combinations. All individuals (*n* = 536) resulted in assignments of mean *q* ^(*i*)^ values of .99 (Figure 2c). In total, 401 individuals were classified as wildcats, 122 as domestic cats, and 13 to different hybrid categories (Table 1). Combining microsatellite and SNP data led to almost identical results as with SNP data alone (Figure 2d).

**Table 1:** Assignment of 1,071 individuals to wildcat, domestic cat, or one of four hybrid categories using NewHybrids. Assignment to west or central population was calculated separately for roadkill or hair trap dataset using Structure *K* = 3

Sample type	Roadkills		Hair traps
Marker type	SNPs (west/central)	Msats (west/central)	Msats (west/central)
Wildcat (WC)	401	387^a^ (201/187)	400 (178/222)
Domestic cat (DC)	122	119^a^ (71/83)	45^a^ (14/59)
First‐generation hybrid (F1)	2 (2/0)	0	1 (0/1)
Second‐generation hybrid (F2)	0	0	1 (0/1)
Backcross to wildcat (F1 × WC)	8^b^ (4/3)	0	1 (0/1)
Backcross to domestic cat (F1 × DC)	3 (1/2)	0	5^a^ (11/0)
Not assigned (*q* ^(*i*)^ < .85)	0	16 (14/8)	44^a^ (45/18)
Total genotyped	536	536	535
Total analyzed in NewHybrids^c^	536	527 (286/278)	516 (248/302)
Total assigned to a parental or a hybrid category with *q* ^(*i*)^ ≥ .85	536	506^a^ (272/270)	453^a^ (203/284)

a Some individuals were analyzed in both the western and central datasets, as they were sampled in the area of overlap of the western and central clusters (Figure S1, see text); in addition, individuals that showed discordant assignments are not included in these totals.

b Four individuals corresponding to the western group, three individuals to the central group, and one individual in the overlapping zone of samples from the central and the western groups.

c Individuals that were not assigned to any of the west or central wildcat clusters (*q* ^(*i*)^ < .75) in the Structure analyses were excluded from the NewHybrids analyses (nine individuals from the roadkill dataset and 19 individuals from the hair dataset).

Assignments using Structure and NewHybrids were mostly consistent (Table S6). In the case of microsatellite‐based analysis, the majority of individuals assigned to potentially parental categories by Structure (*K* = 3) were in agreement with the NewHybrids assignments. However, this agreement was higher for individuals from the central group (98% domestic cats, 99% wildcats) than from the western group (85% domestic cats and 90% wildcats). In the case of hybrid categories, four individuals identified by Structure as domestic cats were classified by NewHybrids as hybrids, and four individuals considered admixed by Structure were assigned to parental species. In the case of SNP‐based analyses, there was one disagreement only (wildcat in Structure, hybrid in NewHybrids).

NewHybrids analyses of roadkill samples based on either microsatellite or SNP data were mostly identical: 99% pure wildcats and 96% domestic cats. Only 13 individuals identified as hybrids based on SNPs were identified as pure wildcats (*n *=* *4) or domestic cats (*n *=* *5) and as not assigned (*n *=* *4) based on microsatellites, resulting in the disagreement of 1% and 4%, respectively (Table S7).

### 3.3 Direction of hybridization

**Figure 3:**
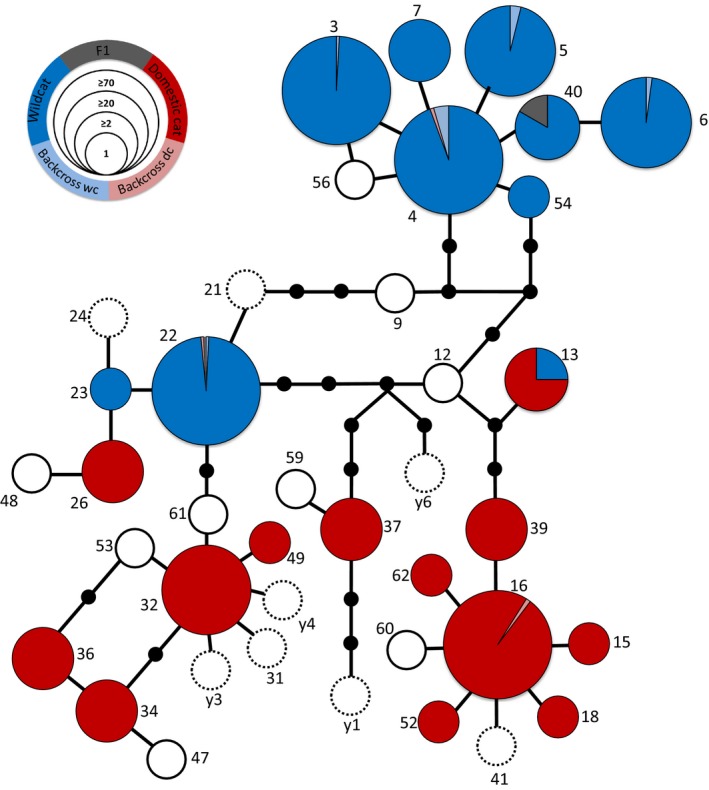
Haplotype network based on data for 1,071 individuals. Small black circles represent haplotypes not found, white circles with a full line were found in the hair dataset, but not in the tissue dataset, and white circles with a dashed line were not observed in this study but obtained from GenBank. The majority of haplotypes are found across entire the study region (FS03/04/16/22/26/32/34/36/37), while a few haplotypes were found only in the western (FS05/07/40) or the central (FS06/23) cluster. Pie chart colors indicate proportion of membership to one of the five groups as determined by SNP‐based NewHybrids analysis, and size of the circles indicate the number of samples analyzed, see inset on top left

We found 22 different haplotypes in the roadkill dataset (Figure 3/Table S8) and 27 in the hair trap samples, of which nine were found in a single hair sample. Five haplotypes (SNG‐HP‐FS03, SNG‐HP‐FS04, SNG‐HP‐FS05, SNG‐HP‐FS06, and SNG‐HP‐FS22) could be assigned to wildcats and were found in 389 individuals. Two haplotypes (SNG‐HP‐16 and SNG‐HP‐32) could be assigned to domestic cats and were found in 93 individuals (SNP data and NewHybrids). The following haplotypes were found in the 36 hybrids identified across all datasets using NewHybrids (Table 1 and Table 2): F1s carried six wildcat, one domestic cat, and four rare haplotypes. The two F2 individuals had two wildcat haplotypes. Backcrosses to wildcats carried 11 wildcat and three domestic cat haplotypes. Individuals identified as backcrosses to domestic cats carried four domestic cat, four wildcat, and two unclassified haplotypes. Migration rates calculated with BayesAss were from wildcats to domestic cats nearly as twice as high than vice versa, ranging between 0.0040 and 0.0131, depending on marker type and population (see Table S9).

**Table 2:** Assignment based on SNP data for individuals that could not be assigned with *q* ^(*i*)^ ≥ .85 using microsatellites in NewHybrids (see Table 1). For the roadkill dataset, the 16 samples are already included in the SNP column of Table 2. From a total of 57 individuals from the hair dataset which could not be clearly assigned (see Table 1), 53 samples were additionally analyzed with SNPs. The discordance between the 44 samples from Table 1 to 57 samples is based on 13 samples that could be assigned in the central NewHybrids run, but could not be clearly assigned in the western NewHybrids run

Sample type	Roadkill (west/central)	Hair traps (west/central)
Wildcat (WC)	8 (5/3)	7 (2/5)
Domestic cat (DC)	4 (3/2)	28 (28/1)
First‐generation hybrid (F1)	1 (1/0)	8 (3/6)
Second‐generation hybrid (F2)	0	1 (1/1)
Backcross to wildcat (F1 × WC)	2 (1/1)	5 (5/1)
Backcross to domestic cat (F1 × DC)	1 (0/1)	2 (2/0)
Not assigned (*q* ^(*i*)^ < .85)	0	2
Total genotyped	16	53

### 3.4 Simulations for hybridization assessment

**Figure 4:**
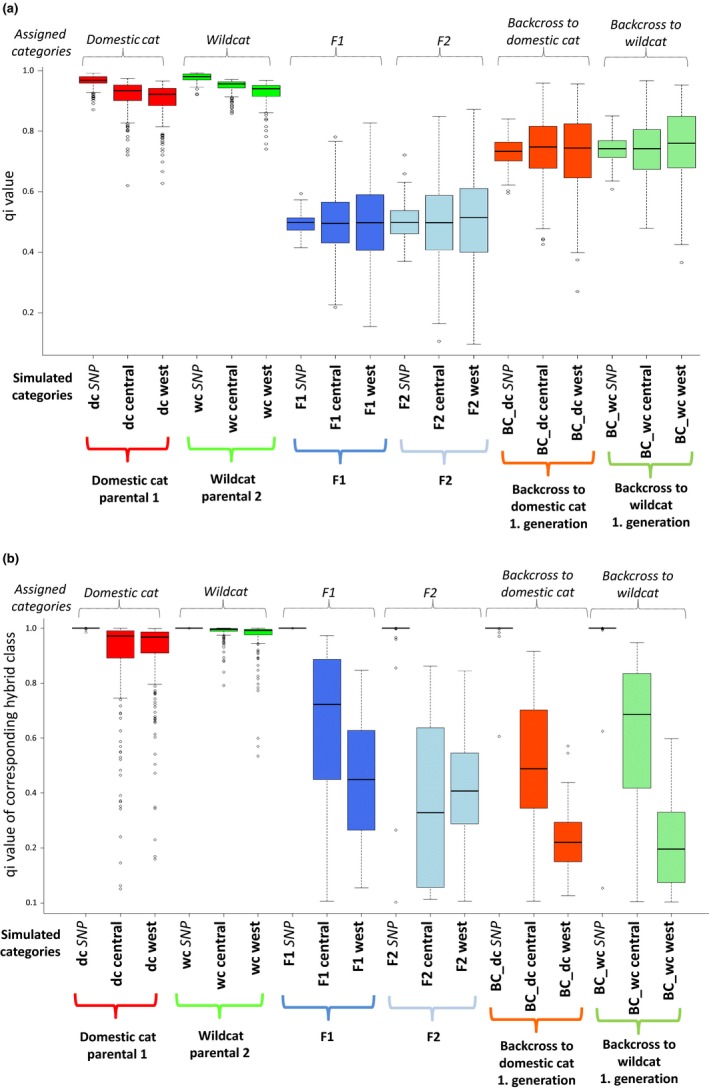
Individual membership values (*q* ^(*i*)^) for wildcats and domestic cats with SNP and microsatellite genotypes generated using Hybridlab and analyzed using Structure (a) and NewHybrids (b). Displayed are also the Median and the *q* ^(*i*)^‐values corresponding to simulated categories

We analyzed the microsatellite genotypes generated using Hybridlab with Structure and NewHybrids in the same way as we did with the empirical data. In the Structure *K* *=* 2 runs, similar results were obtained independent of whether we generated genotypes with domestic cats and either the western or the central wildcat groups as parentals (Figure 4). Using a threshold of *q* ^(*i*)^ ≥ .75, as used earlier, >99.5% of the artificially generated wildcats were assigned to one cluster and >97% of the domestic cats to another cluster (Table S10). Of the simulated F1 hybrids, 2.5% of the genotypes in the central dataset and 8% of the genotypes in the western dataset were incorrectly assigned to either wildcat or domestic cat. In the case of simulated F2s, these results were 9.5% and 10%, respectively. Half of the simulated backcrosses of the first generation (F1 × parental species) were assigned to their respective parental cluster, the other half was not assigned. Backcrosses of the second generation (the progeny of F1 hybrids backcrossed in two successive generations to the same parental group) were in the range 80%–88.5% assigned to the corresponding parental group. Detailed information regarding *q* ^(*i*)^‐values for Structure and NewHybrids runs using simulated genotypes are reported in Tables S10 and S11.

With NewHybrids, the artificially generated microsatellite wildcat genotypes were more reliable assigned to the correct parental category (western group, 96% correct; central group, 99% correct) than for the artificially generated domestic cats (western group, 83% correct; central group, 79% correct; Figure 4 and Figure S2; Tables S12/S13) using a *q* ^(*i*)^ ≥ .85. Only 32% of the simulated F1 hybrids of the central group were correctly assigned by NewHybrids, and all simulated F1 genotypes from the western group could not be assigned with confidence. In the case of the simulated F2s, 98% from the western group and 88% from the central group could not be assigned with confidence. First‐generation backcrosses were mostly not assigned *q* ^(*i*)^ < .85; mean = 79%) and in some cases misclassified (*q* ^(*i*)^ ≥ .85; mean = 10%).

We analyzed the SNP genotypes generated using Hybridlab with Structure and NewHybrids in the same way as described above. Structure results for the simulated dataset using *K = *2 showed that all genotypes from the simulated parental populations were correctly classified. All simulated F1 and F2 genotypes appeared admixed (*q* ^(*i*)^ < .75), and a proportion of 40% of the backcrosses was assigned to each parental cluster (Figure 4; Tables S10/S11). Second‐generation hybrids were assigned with *q* ^(*i*)^ > 99% to each parental cluster, too. NewHybrids assigned all artificially generated pure individuals and F1 hybrids to the correct category with high confidence (*q* ^(*i*)^ ≥ .85; 100%), and almost all artificially generated F2 hybrids, backcrosses to wildcat, and backcrosses to domestic cat were assigned with high confidence (*q* ^(*i*)^ ≥ .85; 96%, 96%, and 98%, respectively) (see Figure 4 and Figure S2; Table S12/S13). Individuals of backcrosses of the second generation were assigned as backcross (*q* ^(*i*)^ ≥ .85; 78% backcross to wildcat or 44% backcross to domestic cat), pure individuals (≥.85; 12% to wildcat or 40% to domestic cat), or were not assigned with confidence (*q* ^(*i*)^ < .85; 10% and 16%, see Table S12).

## 4 DISCUSSION

### 4.1 Hybridization between wildcats and domestic cats in western Central Europe

3.5% of all wildcat individuals collected in western Central Europe were identified as hybrids (11 F1s, two F2s) and backcrosses to both parental groups (14 to wildcat and 10 to domestic cat, Table 1 and 2). Previous studies assessing wildcat and domestic cat hybridization in Central Europe were mostly restricted to a few local populations and reported hybridization rates ranging from 0% to 43%. Pierpaoli et al., (2003) performed the first hybridization analysis of wildcats in Germany, using morphometrics to select domestic cats and wildcats, including 42 samples from western Germany and 33 samples from one location in central Germany. The study found seven potential hybrids, which had been morphologically assigned to domestic cats, but which were admixed according to Structure using 12 microsatellite loci. Interestingly, the carcasses of these cats were collected near human settlements, and four of them had domestic cat food in their stomach (Pierpaoli et al., 2003). Eckert et al., (2010), using samples from morphometrically determined wildcats (*n *=* *63) from six different regions in Germany (three western and three central), reported no evidence of hybridization using seven microsatellite loci and Structure analysis. The highest hybridization rates in Germany were reported in Hertwig et al., (2009), in which 28 cats from the western area and 48 from the central one were genotyped using 11 microsatellite loci and analyzed in Structure with *K *=* *2. They calculated a hybridization rate of 11% over all individuals and indicated that 43% of wildcat individuals from the western and 4% of the central wildcat populations were admixed.

Our study surpasses previous studies in the geographical area covered, the number of samples included, and a genome‐wide SNP panel, encompassing the known distribution of wildcats in Germany and Luxembourg. We found a high number of putative F2s (*n *=* *52; 5%) when samples from the west and the central clusters (as detected by Structure) were analyzed together in NewHybrids. These F2 individuals were mostly assigned to pure wildcats when the western and central clusters (*n *=* *38; 72%) were analyzed separately or with SNP data (for available individuals *n *=* *24; 22 to pure wildcat, one to domestic cat, one to F1). The software NewHybrids assumes any deviation from Hardy–Weinberg equilibrium as originating from hybridization if the allele frequencies of the two taxa prior to hybridization are not known (Anderson & Thompson, 2002), as is the case for wildcats and domestic cats. Because of significant differentiation as indicated by microsatellites between the individuals sampled in the west part and central part of the range (*F*_ST_ = 0.084–0.087 between the western and central clusters, *p* < .001), we analyzed them separately. These results indicated the presence of no hybrids in the western group and two in the central group, but left 60 individuals with assignment probabilities below *q* ^(*i*)^ < .85. SNP data indicated 18 hybrids in the western group and 13 in the central group, which suggests that recent hybridization rates are similarly low in both the western part and central part of the range.

### 4.2 Direction of hybridization

The genetic differentiation between western wildcats and domestic cats is lower than between central wildcats and domestic cats; thus, the occurrence of misassigned individuals affects mostly western wildcats (see our results for all samples analyzed together). The lower differentiation of the western wildcat population and domestic cats was also observed in previous studies (e.g., Cocciararo et al., 2016) and might be due to historic hybridization rather than recent hybridization, supported by the low number of detected hybrids and the generally low migration rates (see Table S9).

The majority of individuals identified as F1 and F2 hybrids and wildcat backcrosses and half of the backcrosses to the domestic cat carried wildcat mtDNA haplotypes, suggesting more mating events between female wildcats and male domestic cats or a hybrid resulting from a cross. This contrasts greatly with the findings of Nussberger, Wandeler, Weber et al., (2014), which concluded that 25% of the individuals identified as wildcats in their study region, using the same SNP marker set, carried a domestic cat haplotype. The analyzed panel from Nussberger, Wandeler, Weber et al., (2014) includes four mtDNA SNPs which were interpreted as being indicative of domestic cat haplotypes. However, in our study, individuals with the SNP allele supposedly indicative of a domestic cat haplotype carried haplotypes SNG‐HP‐FS‐22 and FS‐23 (see Figure 3). These haplotypes are in the haplotype group shared between wildcat and domestic cats, but appear to be private for wildcats based on assignments of these individuals to wildcats based on the autosomal SNP markers and in analyses conducted using NewHybrids. In our case, using these four mtDNA SNPs to identify domestic cat haplotypes would have led us to estimate that 32% of wildcats in our population had domestic cat mtDNA haplotypes. Most haplotypes appeared almost exclusively private in wildcats or domestic cats. None of the individuals identified as pure wildcats using SNPs showed a domestic cat haplotype and *vice versa*.

### 4.3 Methodological aspects of hybridization assessment

The relatively high rate of individuals that could not be assigned to a parental or hybrid category using microsatellite data in NewHybrids (western and eastern clusters run separately) of 6%–11% depending on the priors considered strongly contrasted with a high assignment rate of 0.99 when using the SNP data. The superiority in assignment can be explained by the selection process of the SNP panel, which was based on choosing loci showing greatest *F*_ST_ values between wild and domestic cats (Nussberger et al., 2013). Of those individuals that were assigned with a probability below .85 using microsatellite data, one‐third were characterized as hybrids based on the SNPs, approximately half (44%) as pure domestic cats and the remaining as wildcats (Table S14).

For the SNP dataset, the certainty of assignments was powerful with *q* ^(*i*)^ values higher than .99 for 99% of the individuals. Analyses based on microsatellite data, on the contrary, showed differing numbers of hybrids and varied considerably depending on the combination of sample sets and prior combinations. Our simulations indicated that microsatellite markers could not assign admixed genotypes into the correct hybrid category.

#### 4.3.1 Marker systems

The majority of studies concerning hybridization between domestic and wildcat have relied on microsatellites, mostly 7–13 loci (Beaumont et al., 2001; Eckert et al., 2010; Hertwig et al., 2009; O'Brien, Devillard, & Say, 2009; Oliveira, Godinho, Randi, & Alves, 2008; Oliveira, Godinho, Randi, Ferrand, et al., 2008; Randi, Pierpaoli, Beaumont, Ragni, & Sforzi, 2001; Say et al., 2012), whereas Driscoll, Menotti‐Raymond, & Roca, (2007) and Mattucci et al., (2013) used 36 and 35 loci, respectively. To detect hybridization, knowledge about the relative allelic frequencies of the markers being used in the parental taxa are crucial, and weak discriminatory power to identify hybrids beyond the first generation is common, as it has been shown using Bayesian approaches for simulated data of microsatellites (Vähä & Primmer, 2006), or by simulating hybrid individuals using empirical microsatellite data from wildcat and domestic cat parentals (Hertwig et al., 2009; O'Brien et al., 2009; Oliveira, Godinho, Randi, & Alves, 2008). Many studies about wildcat hybridization used simulated data to calculate a significant threshold based on their data and used *q* ^(*i*)^ values higher than .95 to select the individuals that made the parental populations (Hertwig et al., 2009; Nussberger et al., 2013; Say et al., 2012).

Our results based on simulated genotypes support the power of microsatellites to distinguish purebred from admixed individuals up to the first hybrid generation using NewHybrids (no wrongly assigned F1 individuals, but 84% with *q* ^(*i*)^ < .85). Microsatellites did not allow, however, to differentiate between hybrid classes, including backcrosses. In contrast, our simulated SNP genotypes were assigned with high certainty to parental and hybrid categories up to the second hybrid generation (a mean value of 12% per category was wrongly assigned, Table S12). Thus, the recently developed SNP set appears as a promising tool for identifying hybrids of different categories in wildcats. However, the success in identifying simulated hybrids is limited beyond the second generation, concording with (Nussberger et al., 2013). For studying ancient hybridization of wildcats and domestic cats that may have been going on since Roman times, it may be appropriate to focus on whole genomes in of future studies.

#### 4.3.2 Sampling strategy

Based on the 27 hybrids, the hybridization rate, here defined as proportion of F1, F2, and wildcat backcrosses in relation to the total number of identified wildcats and excluding the backcrosses to domestic cat following Nussberger, Wandeler, Weber et al. (2014), is 3.4%. When only considering F1 hybrids, the hybridization rate is 1.4%. Interestingly, the sampling strategy revealed differing hybridization rates of 3.9% for the hair trap dataset and 2.4% for the roadkill samples. In contrast to the roadkill dataset, among which only two F1 individuals were detected, the hair dataset revealed nine F1 individuals. We cannot explain whether these findings originate from differing sampling techniques alone or if they actually reflect differences in occurrences of hybrids. A possible explanation is a sampling bias toward morphologically wildcat‐like individuals in the case of roadkills, which would decrease the number of potential hybrids in the roadkill dataset, including F1s.

Our study shows that different methodological approaches may lead to different conclusions regarding hybridization rates between wild cats and domestic cats. In particular, the choice of markers as well as the sampling strategy may impact the conclusions regarding hybrid frequencies.

### 4.4 Factors driving hybridization

Identifying factors driving hybridization is important to develop strategies for conservation like, for example, the identification of management units for ongoing plans to reconnect isolated populations or to implement measures that reduce the risk of extinction through the loss of genetic integrity. The low hybridization rates found in Central European wildcats might be due to several reasons. In telemetry studies conducted in western Germany, wildcats have been shown to generally avoid human settlements (Klar, Fernández, & Kramer‐Schadt, 2008). The lower hybridization rates found in this study compared to studies from other European regions might thus be due to the persistence of large forest fragments in the German low‐mountain region, reducing the contact between wild and domestic cats. In northeastern France, Germain, Benhamou, & Poulle, (2008) showed that wildcats, domestic cats, and hybrids, identified based on morphology and genetic markers, had nearly the same activity rhythm but low spatial overlap of home ranges. They concluded that hybridization may be driven by rare events in which either wildcats or domestic cats ventured into each other's territories (forest or farms, respectively), or by the less strong spatial separation between hybrids and their parentals than between wildcats and domestic cats, in which case backcrosses to either wildcats or domestic cats would be favored (rather than F1s). Moreover, territoriality and intraspecific aggressive interactions are common in wild felid species (e.g., Mattisson, Persson, Andren, & Segerstrom, 2011; Sunquist & Sunquist, 2002), including wildcats (Piechocki, 1990), whereas domestic cats show a substantial degree of intraspecific sociality or communality (Bradshaw, 2016), which may lead to domestic cats being expelled from wildcat habitat.

Nevertheless, at least some local and temporal coexistence of domestic cats and wildcats has been observed, including in Germany (Steyer et al., 2013), and also in Portugal (Sarmento, 1996; Sarmento, Cruz, Eira, & Fonseca, 2009), Spain (Gil‐Sanchez, Jaramillo, & Barea‐Azcon, 2015), Switzerland (Nussberger, Wandeler, Weber et al., 2014), and Italy (Anile, Arrabito, Mazzamuto, Scornavacca, & Ragni, 2012). Particularly in regions with highly fragmented habitat, coexistence is expected to be more common, as wildcats may venture into human‐associated habitat where domestic cats are more abundant. When wildcat numbers are reduced or in the expanding front of a recolonizing wildcat population, hybridization may be more common due to difficulties in finding appropriate mates due to density effects, such as Allee effects (Allee, 1931). A situation in which individuals of the diminished species mate with individuals of the other, more abundant, species has been reported in other mammals, such as wolves in British Columbia (Muñoz‐Fuentes et al., 2010) or gazelles in Central Africa (Godinho, Abáigar, & Lopes, 2012). Concordant with this, higher hybridization rates at the edge of wildcat distribution areas has been described in Italy (Randi, 2008) and Switzerland (Nussberger, Wandeler, Weber et al., 2014). In our study and in a previous one in France (Say et al., 2012), a concentration of hybrids near the edge of the distribution was not apparent, but that could also be due to the overall low number of detected hybrids. Future projects, including samples from France and Belgium, will allow further insights into hybridization processes.

### 4.5 Hybridization and wildcat conservation

**Figure 5:**
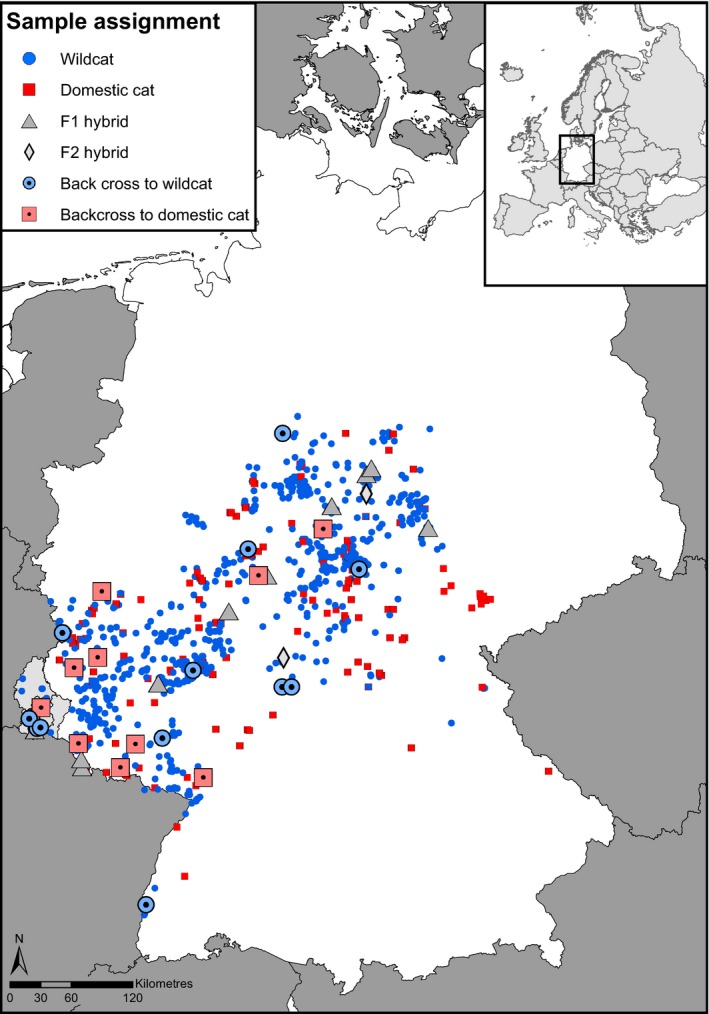
Map of Germany (white) and Luxembourg (light gray) showing geographical locations for all samples and their assignment to wildcats, domestic cats, or their hybrids according to NewHybrids analysis based on SNP and (in case of unavailability of SNPs) microsatellite data (*n*_wildcat_ = 801; *n*_domestic cat_ = 195; *n*_F1_ = 11; *n*_F2_ = 2; *n*_backcross to wildcat_ = 14; *n*_backcross to domestic cat_ = 10)

The impact of hybridization on wildlife is diverse, ranging from genetic extinction in various fish, mammal, and bird species (Rhymer & Simberloff, 1996), to even increased evolutionary potentials in, for example, sunflowers (Kim & Rieseberg, 1999) or Soay sheep (Feulner, Gratten, & Kijas, 2013). Introgression of domestic traits into the wildcat population may have adaptive consequences. A comparative whole‐genome study on domestic cats and wildcats revealed positive selection on genes associated with diet, sensory processes, behavior, and reward in domestic cats (Montague, Li, & Gandolfi, 2014). Moreover, Monzon, Kays, & Dykhuizen, (2014) suggest that introgression of domestic dog alleles in coyotes could have led to adaptations to more human‐dominated environments, which might be a positive aspect of such hybridization events from domestic traits into the wild forms. Regular monitoring of hybridization levels in wildcat populations is important, in particular in relation to already built and current plans to build green corridors for achieving a nationwide biotope network (Cocciararo et al., 2016; Vogel & Mölich, 2013). The finding that overall hybridization rate is low has important consequences for ongoing efforts to reconnect fragmented forest habitats in Germany and to form a network of forest corridors allowing wildcats and other species to disperse between habitat patches (Vogel & Mölich, 2013). In contrast to earlier studies, our results imply that there are no regions with high hybridization rates that would require to be kept isolated (Figure 5). Thus, we see no contraindication to reconnect the entire wildcat distribution range in our study region. The application of the SNP chip (Nussberger, Wandeler, & Camenisch, 2014) to identify hybrids seems most appropriate for this task, due to the high resolution of different hybrid categories. In addition, SNP chips have methodological advantages over microsatellites, such as decreased subjectivity in scoring, reduced price, and fast processing of large number of samples (de Groot, Nowak, Skrbinšek et al., 2016).

## 5 CONCLUSION

The low rate of hybridization and introgression of domestic cat alleles in the Central European wildcat genepool (3.5% or 1.4% F1 hybrids) appears somewhat surprising, given the fact that Germany and Luxembourg are densely populated areas with highly fragmented habitats for the species. Despite that wildcats are outnumbered by domestic cats by a factor of at least 10^3^, hybridization seems to be a rare event and no signs of recent domestic cat introgression can be found in most wildcats analyzed. This raises confidence in the potential for successful long‐term maintenance of wildcats even in densely populated, highly anthropogenically altered landscapes. Together with the recently observed stunning return of large carnivores in Europe (Chapron, Kaczensky, & Linnell, 2014) and other species, humans and elusive wildlife may indeed successfully coexist in areas such as western and Central Europe, where no primeval forests or other types of wilderness‐like habitats have persisted to present times.

## CONFLICT OF INTEREST

None declared.

## AUTHOR CONTRIBUTIONS

All authors conceptualized this study; A.T. and K.S. performed the molecular laboratory work and analyzed the data with V.M.F.; A.T. and K.S. wrote the first draft, with major contribution from V.M.F.; all authors contributed to the final version of the manuscript; C.N. acquired funding. All authors read and approved the current version of the article.

## DATA ACCESSIBILITY

Sampling locations, all genetic raw data, and results from the software analyses as reported in this study, including mitochondrial sequences, genotypes from microsatellites, and SNPs, are available via Dryad (https://doi.org/10.5061/dryad.fp954). DNA sequence data used for haplotype network have been submitted to GenBank and have accession numbers KX161418‐KX161423. Additional analyses are provided as supporting information.
