# Supplementary material for: Low rates of hybridization between European wildcats and domestic cats in a human‐dominated landscape
Source: Ecol Evol. 2018 Jan 27;8(4):2290–304. doi: 10.1002/ece3.3650 (PMC5817136; doi:10.1002/ece3.3650)
Supplement: Supplementary file 1 [file ECE3-8-2290-s001.doc]

# Electronic supplementary material

Steyer, K., Tiesmeyer, A., Muñoz-Fuentes, V., Nowak, C. (2017) Low rates of hybridization between European wildcats and domestic cats in a human-dominated landscape

[Figure S 1 Definition of the overlap area for the two wildcat clusters as identified by Structure based on the genotypes corresponding to 1138 individuals for hair samples collected with the lure stick method and genotyped for the same 14 microsatellite markers as described in this paper (Steyer et al. 2016 “Large-scale genetic census of an elusive carnivore, the European Wildcat Felis s. silvestris”). Each dot represents an individual and the color, blue or yellow, denotes assignment to one of the two wildcat clusters with q^(i)^≥0.75 (Structure run for K=3, domestic cats are not shown). 3](#__RefHeading___Toc488418609)

[Figure S 2 Assignment of parental and hybrid genotypes generated using Hybridlab and analysed with the software NewHybrids. 4](#__RefHeading___Toc488418610)

[Table S 1 Samples of wildcats and domestic cats used in this study. Sampling year, location and sample type are indicated. 5](#__RefHeading___Toc488418595)

[Table S 2 List of haplotypes and frequency for the roadkill (n=536) and hair trap (n=535) dataset. 5](#__RefHeading___Toc488418596)

[Table S 3 F_ST_ values for wildcats and domestic cats. WC, wildcat; ***P<0.001 7](#__RefHeading___Toc488418597)

[Table S 4 Allele frequencies for 60 SNPs in wildcats and domestic cats sampled in Luxembourg and Germany for the three clusters as identified by Structure based on 14 microsatellites (see text for details). Genotypes not clearly assigned to any of the three clusters (q^(i)^<0.75) were excluded here (n=24). WC west, wildcats sampled in Luxembourg and western Germany; WC central, wildcats sampled in central Germany; DC, domestic cats. 7](#__RefHeading___Toc488418598)

[Table S 5 Microsatellite allele frequencies for wildcats and domestic cats sampled in Luxembourg and Germany for the three clusters as identified by Structure. Genotypes not clearly assigned to any of the three clusters (q^(i)^<0.75) were excluded from this list (n=24 and n=39 for the roadkill and the hair trap datasets, respectively). WC_west, wildcats sampled in Luxembourg and western Germany; WC_central, wildcats sampled in central Germany; DC, domestic cats. Alleles in bold were only found in one of the two subspecies in that sample dataset. 11](#__RefHeading___Toc488418599)

[Table S 6 Values represent absolute numbers of assignment of individuals using Structure and NewHybrids. All hybrid categories in NewHybrids are summed up to one hybrid group. For the hair trap dataset 19 individuals which could not be assigned to any of the west or central wildcat clusters (q^(i)^<0.75) in the STRUCTURE analyses were excluded; also 19 individuals which were analyzed in both the western and central dataset, which were sampled in the area of overlap of the western and central clusters, were excluded. 15](#__RefHeading___Toc488418600)

[Table S 7 Comparison of parental or hybrid categories for the roadkill dataset using NewHybrids. Disagreements are shown as percentage of individuals with differing classifications. Individuals that could not be assigned to any of the three clusters (wc_west, wc_central, domestic cats) were excluded (n=9). Five individuals indicated within brackets were collected in the area of overlap of the western and central clusters and were, therefore, run in both microsatellite NewHybrids runs but could only be assigned to a category with q^(i)^<0.85 in one run. 15](#__RefHeading___Toc488418601)

[Table S 8 Assignment of haplotypes to wildcat or domestic cat based on NewHybrids analysis. Individuals that were not assigned to any of the west or central wildcat clusters (q^(i)^<0.75) in the Structure analyses were excluded from the NewHybrids analyses (9 individuals from the roadkill dataset). 16](#__RefHeading___Toc488418602)

[Table S 9 Migration rates assessed using the software BayesAss (Wilson and Rannala 2003). Shown values are the number of individuals of the first named group that derived from the other group per generation. Standard deviation of marginal posterior distribution is shown in brackets. 16](#__RefHeading___Toc488418603)

[Table S 10 Genotypes generated using Hybridlab and assigned with Structure. The numbers are percentage of individuals (n=200) assigned to the respective cluster. 18](#__RefHeading___Toc488418604)

[Table S 11 Mean q^(i)^-values for Structure and ranges of simulated genotypes for their respective hybrid category are shown. Rates of not classified genotypes are reported. Q^(i)^-values above 0.75 are represented as clearly assigned. 19](#__RefHeading___Toc488418605)

[Table S 12 Genotypes generated with Hybridlab and assigned with NewHybrids. The numbers are percentage of individuals (n=200) assigned to the respective cluster. 20](#__RefHeading___Toc488418606)

[Table S 13 Mean q^(i)^ values and ranges of simulated genotypes for their respective hybrid category for the NewHybrids results. Q^(i)^-values above 0.85 was used as a threshold with our empirical data set. 21](#__RefHeading___Toc488418607)

[Table S 14 Samples in NewHybrids with q^(i)^<0.85 using microsatellites, showing their corresponding SNP and mtDNA results. Parental wildcat= WC; parental domestic cat= DC; first generation hybrid= F1; second generation hybrid= F2; backcross to parental wildcat= BC_WC; backcross to parental domestic cat= BC_DC; not run= sample was not analysed with SNPs; bad quality= SNP analyses could not be performed due to low number of amplified SNP loci. 22](#__RefHeading___Toc488418608)

# Supplementary Figures


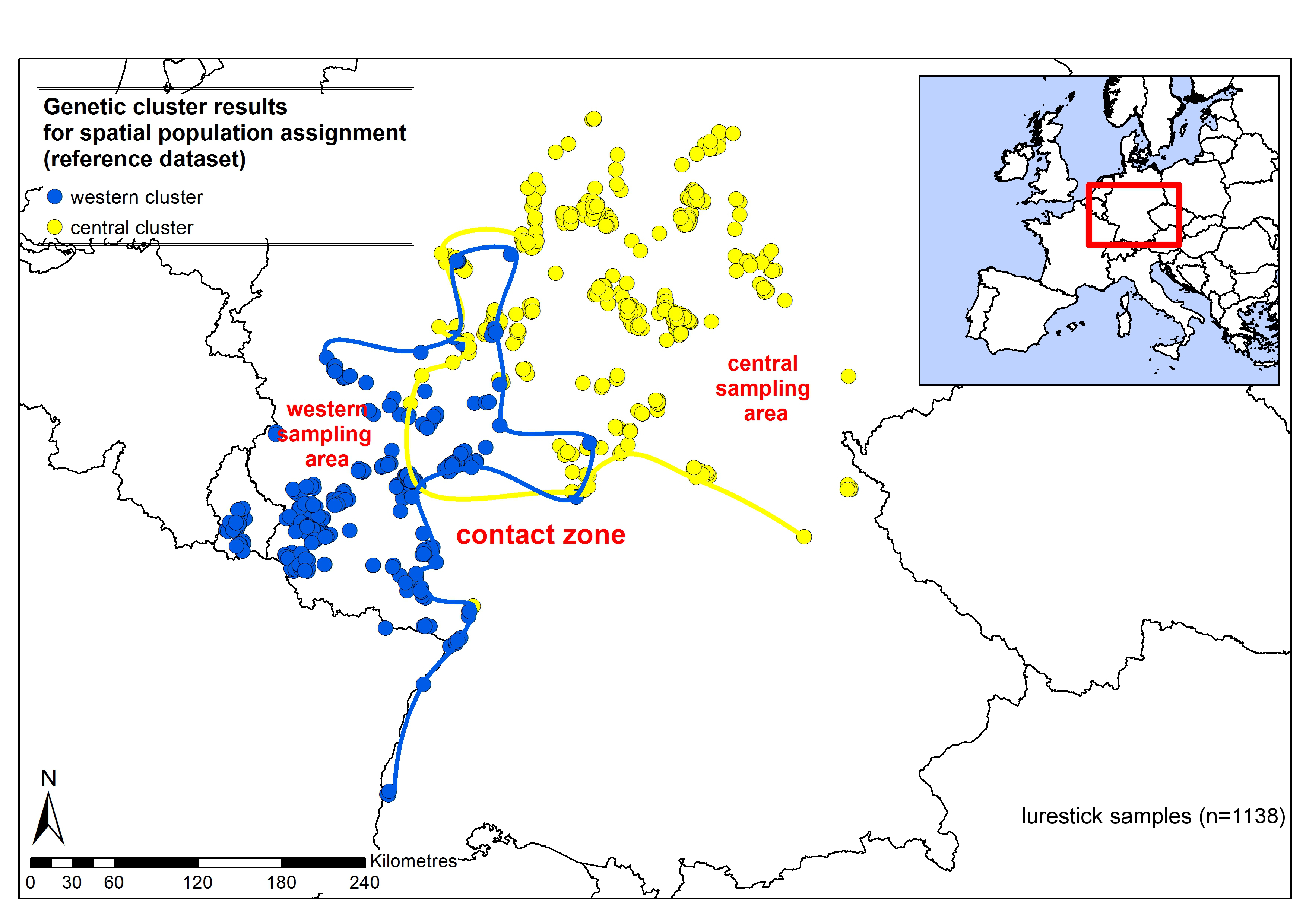


Figure S 1 Definition of the overlap area for the two wildcat clusters as identified by **Structure** based on the genotypes corresponding to 1138 individuals for hair samples collected with the lure stick method and genotyped for the same 14 microsatellite markers as described in this paper (Steyer et al. 2016 “Large-scale genetic census of an elusive carnivore, the European Wildcat *Felis s. silvestris*”). Each dot represents an individual and the color, blue or yellow, denotes assignment to one of the two wildcat clusters with *q^(i)^*≥0.75 (**Structure** run for *K*=3, domestic cats are not shown).


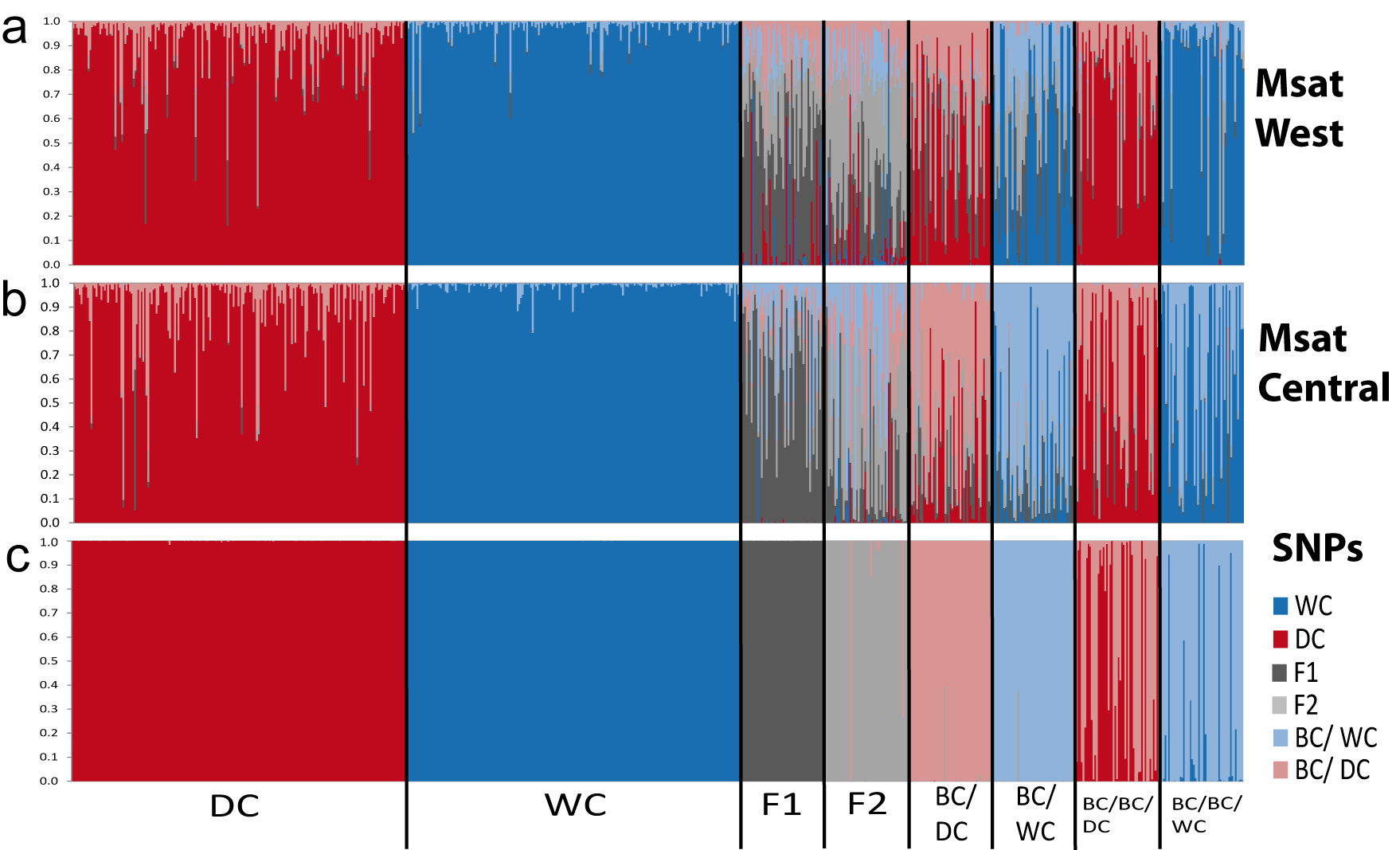


Figure S 2 Assignment of parental and hybrid genotypes generated using **Hybridlab** and analysed with the software **NewHybrids**.

# Supplementary Tables

Table S 1 Samples of wildcats and domestic cats used in this study. Sampling year, location and sample type are indicated.

|  | Germany |  |  |  | Luxembourg |  |
| --- | --- | --- | --- | --- | --- | --- |
| **year** | **blood samples** | **tissue samples** | **hair samples** |  | **tissue samples** | **hair samples** |
| **1995** |  | 3 |  |  |  |  |
| **1996** |  | 7 |  |  |  |  |
| **1997** |  | 13 |  |  |  |  |
| **1998** |  | 6 |  |  |  |  |
| **1999** |  | 13 |  |  |  |  |
| **2000** |  | 14 |  |  |  |  |
| **2001** |  | 1 |  |  | 1 |  |
| **2002** | 1 | 2 |  |  |  |  |
| **2003** | 3 | 11 |  |  | 1 |  |
| **2004** | 2 | 10 |  |  |  |  |
| **2005** | 1 | 21 |  |  | 2 |  |
| **2006** | 1 | 29 |  |  | 1 |  |
| **2007** |  | 17 | 2 |  | 1 |  |
| **2008** | 27 | 23 |  |  | 3 |  |
| **2009** |  | 62 | 19 |  | 6 | 1 |
| **2010** | 3 | 75 | 147 |  | 1 | 13 |
| **2011** | 2 | 65 | 68 |  |  | 42 |
| **2012** | 5 | 52 | 522 |  |  | 14 |
| **2013** | 3 | 45 | 194 |  |  |  |
| **2014** |  | 3 |  |  |  |  |
| **total** | **48*** | **472** | **952** |  | **16** | **70** |

(*one blood sample was obtained from a fresh roadkill)

Table S 2 List of haplotypes and frequency for the roadkill (n=536) and hair trap (n=535) dataset.

| **SNG-HP-FS-** |  | **GenBank accession numbers^1)^** | **n_roadkill_/n_hair trap_** |
| --- | --- | --- | --- |
| **03** |  | KR076400; EU313863; GQ268269; GQ268270; GQ268298; GQ268306; GQ268314; GQ268316; JX045658 | (111/109) |
| **04** |  | KR076401; GQ268315; EU313829; EU313849; GQ268313; EU313830; EU313855; EU313859; GQ268287; GQ268312; GQ268311; GQ268310; GQ268297 | (89/99) |
| **05** |  | KR076402; EU313833; EU313850 | (26/10) |
| **06** |  | KR076403 | (47/40) |
| **07** |  | KR076404; GQ268309 | (4/2) |
| **09** |  | KR076405 | (0/1) |
| **12** |  | KR076406; EU313817; EU313870 | (0/3) |
| **13** |  | KR076407; EU313857; GQ268250; GQ268257; GQ268260; EU313857 | (4/2) |
| **15** |  | KR076408 | (1/1) |
| **16** |  | KR076409; GQ268240; GQ268246; GQ268241; GQ268247; GQ268238; GQ268248; EU313867; EU313865; GQ268243; GQ268251; GQ268237; GQ268258; EU313862; EU313854; EU313847; GQ268259; GQ268261; EU313838; EU313837; EU313836; EU313825; EU313824; GQ268263; JX045661; EU313823; GQ268266 | (74/56) |
| **18** |  | KX161418 | (1/0) |
| **21** |  | KR076410 | - |
| **22** |  | KR076411; GQ268278; GQ268279; GQ268277; GQ268280; GQ268282; GQ268283; GQ268284; GQ268274; GQ268275; GQ268271; GQ268299; GQ268290; GQ268291; GQ268293; GQ268295; GQ268303; GQ268302; GQ268304; GQ268307; GQ268308; GQ268244; GQ268233; JX045660 | (127/150) |
| **23** |  | KR076412; EU313858; EU313840 | (1/7) |
| **24** |  | KR076413; EU313846; EU313839; EU313842 | - |
| **26** |  | KR076414; EU313861; EU313826; GQ268249; GQ268264; EU313860; | (3/7) |
| **31** |  | KR076415; EU313822; GQ268256 | - |
| **32** |  | KR076416; GQ268239; GQ268236; GQ268235; EU313821; EU313818; GQ268245; GQ268267; GQ268262; GQ268253; GQ268255; GQ268265; EU313845 | (20/10) |
| **34** |  | KR076417; GQ268254; EU313864; GQ268252 | (7/5) |
| **36** |  | KR076418; GQ268242; EU313856; EU313871 | (5/5) |
| **37** |  | KR076419; EU313816; GQ268268; EU313843; U20753_2; U20753; GQ268234 | (4/2) |
| **39** |  | KR076420 | (2/0) |
| **40** |  | KR076421; EU313851; EU313831; EU313841 | (6/17) |
| **41** |  | KR076422; EU313853 | - |
| **47** |  | KR076423; EU313872 | (0/1) |
| **48** |  | KR076424 | (0/1) |
| **49** |  | KX161419 | (1/0) |
| **52** |  | KR076425 | (1/1) |
| **53** |  | KR076426; EU313866 | (0/1) |
| **54** |  | KR076427; EU313848; EU313834 | (1/0) |
| **56** |  | KR07642 | (0/2) |
| **59** |  | KX161420 | (0/1) |
| **60** |  | KX161421 | (0/1) |
| **61** |  | KX161422 | (0/1) |
| **62** |  | KX161423 | (1/0) |
| **y1** |  | EU313835 | - |
| **y3** |  | EU313869; EU313868 | - |
| **y4** |  | EU313820; EU313844 | - |
| **y6** |  | EU313852 | - |

^1)^ Our haplotypes can have multiple accession numbers because the published sequences are normally longer 28 than the fragment used here (110bp), and thus can differ at positions outside our target fragment

Table S 3 *F_ST_* values for wildcats and domestic cats. WC, wildcat; ***P<0.001

| **Hair traps, Msats** | | | |  | **Roadkills, Msats** | | | |
| --- | --- | --- | --- | --- | --- | --- | --- | --- |
|  | **WC west** | **WC central** | **Domestic cat** |  |  | **WC west** | **WC central** | **Domestic cat** |
| **WC west** | - |  |  |  | **WC west** | - |  |  |
| **WC central** | 0.087*** | - |  |  | **WC central** | 0.084*** | - |  |
| **Domestic cat** | 0.094*** | 0.170*** | - |  | **Domestic cat** | 0.091*** | 0.162*** | - |
|  |  |  |  |  |  |  |  |  |
| **Roadkills, SNPs** | | | |  | | | | |
|  | **WC west** | **WC central** | **Domestic cat** |  | | | | |
| **WC west** | - |  |  |  | | | | |
| **WC central** | 0.066*** | - |  |  | | | | |
| **Domestic cat** | 0.783*** | 0.803*** | - |  | | | | |
|  |  |  |  |  | | | | |

Table S 4 Allele frequencies for 60 SNPs in wildcats and domestic cats sampled in Luxembourg and Germany for the three clusters as identified by **Structure** based on 14 microsatellites (see text for details). Genotypes not clearly assigned to any of the three clusters (*q^(i)^*<0.75) were excluded here (*n=*24). WC west, wildcats sampled in Luxembourg and western Germany; WC central, wildcats sampled in central Germany; DC, domestic cats.

|  |  |  | **Roadkills (*n=*536)** | | |
| --- | --- | --- | --- | --- | --- |
| **Locus** | **Allele** |  | **WC west** | **WC central** | **DC** |
| ***n*** |  |  | 199 | 189 | 124 |
| **GTA0001768** | 1 |  | 0.051 | 0.003 | 0.931 |
|  | 2 |  | 0.949 | 0.997 | 0.069 |
|  |  |  |  |  |  |
| **GTA0001786** | 1 |  | 0.086 | 0.005 | 0.963 |
|  | 2 |  | 0.914 | 0.995 | 0.037 |
|  |  |  |  |  |  |
| **GTA0017675** | 1 |  | 0.191 | 0.259 | 0.996 |
|  | 2 |  | 0.809 | 0.741 | 0.004 |
|  |  |  |  |  |  |
| **GTA0017683** | 1 |  | 0.035 | 0.005 | 0.793 |
|  | 2 |  | 0.965 | 0.995 | 0.207 |
|  |  |  |  |  |  |
| **GTA0017691** | 1 |  | 0.015 | 0.003 | 0.908 |
|  | 2 |  | 0.985 | 0.997 | 0.092 |
|  |  |  |  |  |  |
| **GTA0017708** | 1 |  | 0.035 | 0.005 | 0.605 |
|  | 2 |  | 0.965 | 0.995 | 0.395 |
|  |  |  |  |  |  |
| **GTA0001769** | 1 |  | 0.025 | 0.138 | 0.919 |
|  | 2 |  | 0.975 | 0.862 | 0.081 |
|  |  |  |  |  |  |
| **GTA0001778** | 1 |  | 0.120 | 0.101 | 0.840 |
|  | 2 |  | 0.880 | 0.899 | 0.160 |
|  |  |  |  |  |  |
| **GTA0001787** | 1 |  | 0.047 | 0.022 | 0.975 |
|  | 2 |  | 0.953 | 0.978 | 0.025 |
|  |  |  |  |  |  |
| **GTA0017652** | 1 |  | 0.109 | 0.077 | 0.806 |
|  | 2 |  | 0.891 | 0.923 | 0.194 |
|  |  |  |  |  |  |
| **GTA0017660** | 1 |  | 0.036 | 0.008 | 0.560 |
|  | 2 |  | 0.964 | 0.992 | 0.440 |
|  |  |  |  |  |  |
| **GTA0017668** | 1 |  | 0.071 | 0.013 | 0.823 |
|  | 2 |  | 0.929 | 0.987 | 0.177 |
|  |  |  |  |  |  |
| **GTA0001770** | 1 |  | 0.037 | 0.022 | 0.939 |
|  | 2 |  | 0.963 | 0.978 | 0.061 |
|  |  |  |  |  |  |
| **GTA0001779** | 1 |  | 0.023 | 0.011 | 0.867 |
|  | 2 |  | 0.977 | 0.989 | 0.133 |
|  |  |  |  |  |  |
| **GTA0017653** | 1 |  | 0.015 | 0.040 | 0.762 |
|  | 2 |  | 0.985 | 0.960 | 0.238 |
|  |  |  |  |  |  |
| **GTA0017661** | 1 |  | 0.088 | 0.027 | 0.851 |
|  | 2 |  | 0.912 | 0.973 | 0.149 |
|  |  |  |  |  |  |
| **GTA0017677** | 1 |  | 0.038 | 0.000 | 0.839 |
|  | 2 |  | 0.962 | 1.000 | 0.161 |
|  |  |  |  |  |  |
| **GTA0017685** | 1 |  | 0.015 | 0.008 | 0.832 |
|  | 2 |  | 0.985 | 0.992 | 0.168 |
|  |  |  |  |  |  |
| **GTA0017701** | 1 |  | 0.246 | 0.479 | 0.935 |
|  | 2 |  | 0.754 | 0.521 | 0.065 |
|  |  |  |  |  |  |
| **GTA0017710** | 1 |  | 0.013 | 0.008 | 0.694 |
|  | 2 |  | 0.987 | 0.992 | 0.306 |
|  |  |  |  |  |  |
| **GTA0001771** | 1 |  | 0.005 | 0.032 | 0.923 |
|  | 2 |  | 0.995 | 0.968 | 0.077 |
|  |  |  |  |  |  |
| **GTA0001780** | 1 |  | 0.058 | 0.013 | 0.859 |
|  | 2 |  | 0.942 | 0.987 | 0.141 |
|  |  |  |  |  |  |
| **GTA0017662** | 1 |  | 0.008 | 0.005 | 0.899 |
|  | 2 |  | 0.992 | 0.995 | 0.101 |
|  |  |  |  |  |  |
| **GTA0017670** | 1 |  | 0.010 | 0.005 | 0.867 |
|  | 2 |  | 0.990 | 0.995 | 0.133 |
|  |  |  |  |  |  |
| **GTA0017686** | 1 |  | 0.022 | 0.009 | 0.822 |
|  | 2 |  | 0.978 | 0.991 | 0.178 |
|  |  |  |  |  |  |
| **GTA0017702** | 1 |  | 0.015 | 0.000 | 0.900 |
|  | 2 |  | 0.985 | 1.000 | 0.100 |
|  |  |  |  |  |  |
| **GTA0018193** | 1 |  | 0.026 | 0.008 | 0.899 |
|  | 2 |  | 0.974 | 0.992 | 0.101 |
|  |  |  |  |  |  |
| **GTA0018206** | 1 |  | 0.019 | 0.009 | 0.763 |
|  | 2 |  | 0.981 | 0.991 | 0.237 |
|  |  |  |  |  |  |
| **GTA0001790** | 1 |  | 0.020 | 0.011 | 0.940 |
|  | 2 |  | 0.980 | 0.989 | 0.060 |
|  |  |  |  |  |  |
| **GTA0017663** | 1 |  | 0.070 | 0.017 | 0.781 |
|  | 2 |  | 0.930 | 0.983 | 0.219 |
|  |  |  |  |  |  |
| **GTA0017687** | 1 |  | 0.171 | 0.005 | 0.948 |
|  | 2 |  | 0.829 | 0.995 | 0.052 |
|  |  |  |  |  |  |
| **GTA0017695** | 1 |  | 0.023 | 0.013 | 0.899 |
|  | 2 |  | 0.977 | 0.987 | 0.101 |
|  |  |  |  |  |  |
| **GTA0017703** | 1 |  | 0.038 | 0.016 | 0.859 |
|  | 2 |  | 0.962 | 0.984 | 0.141 |
|  |  |  |  |  |  |
| **GTA0001773** | 1 |  | 0.016 | 0.008 | 0.879 |
|  | 2 |  | 0.984 | 0.992 | 0.121 |
|  |  |  |  |  |  |
| **GTA0001783** | 1 |  | 0.028 | 0.013 | 0.926 |
|  | 2 |  | 0.972 | 0.987 | 0.074 |
|  |  |  |  |  |  |
| **GTA0001791** | 1 |  | 0.019 | 0.007 | 0.929 |
|  | 2 |  | 0.981 | 0.993 | 0.071 |
|  |  |  |  |  |  |
| **GTA0017672** | 1 |  | 0.013 | 0.040 | 0.790 |
|  | 2 |  | 0.987 | 0.960 | 0.210 |
|  |  |  |  |  |  |
| **GTA0017688** | 1 |  | 0.027 | 0.129 | 0.731 |
|  | 2 |  | 0.973 | 0.871 | 0.269 |
|  |  |  |  |  |  |
| **GTA0017705** | 1 |  | 0.065 | 0.106 | 0.907 |
|  | 2 |  | 0.935 | 0.894 | 0.094 |
|  |  |  |  |  |  |
| **GTA0001774** | 1 |  | 0.261 | 0.048 | 0.908 |
|  | 2 |  | 0.739 | 0.952 | 0.092 |
|  |  |  |  |  |  |
| **GTA0001784** | 1 |  | 0.020 | 0.005 | 0.907 |
|  | 2 |  | 0.980 | 0.995 | 0.093 |
|  |  |  |  |  |  |
| **GTA0017649** | 1 |  | 0.013 | 0.013 | 0.711 |
|  | 2 |  | 0.987 | 0.987 | 0.289 |
|  |  |  |  |  |  |
| **GTA0017681** | 1 |  | 0.020 | 0.043 | 0.594 |
|  | 2 |  | 0.980 | 0.957 | 0.407 |
|  |  |  |  |  |  |
| **GTA0017689** | 1 |  | 0.013 | 0.011 | 0.646 |
|  | 2 |  | 0.987 | 0.989 | 0.354 |
|  |  |  |  |  |  |
| **GTA0017697** | 1 |  | 0.076 | 0.011 | 0.777 |
|  | 2 |  | 0.924 | 0.989 | 0.223 |
|  |  |  |  |  |  |
| **GTA0017706** | 1 |  | 0.135 | 0.095 | 0.730 |
|  | 2 |  | 0.865 | 0.905 | 0.270 |
|  |  |  |  |  |  |
| **GTA0018209** | 1 |  | 0.035 | 0.014 | 0.832 |
|  | 2 |  | 0.965 | 0.986 | 0.168 |
|  |  |  |  |  |  |
| **GTA0001776** | 1 |  | 0.055 | 0.066 | 0.956 |
|  | 2 |  | 0.945 | 0.934 | 0.044 |
|  |  |  |  |  |  |
| **GTA0001785** | 1 |  | 0.122 | 0.005 | 0.931 |
|  | 2 |  | 0.878 | 0.995 | 0.069 |
|  |  |  |  |  |  |
| **GTA0017650** | 1 |  | 0.200 | 0.003 | 0.898 |
|  | 2 |  | 0.800 | 0.997 | 0.102 |
|  |  |  |  |  |  |
| **GTA0017690** | 1 |  | 0.053 | 0.024 | 0.465 |
|  | 2 |  | 0.947 | 0.976 | 0.535 |
|  |  |  |  |  |  |
| **GTA0018198** | 1 |  | 0.386 | 0.335 | 0.645 |
|  | 2 |  | 0.614 | 0.665 | 0.355 |
|  |  |  |  |  |  |
| **id_GTA0017699** | 1 |  | 0.010 | 0.008 | 0.595 |
|  | 2 |  | 0.990 | 0.992 | 0.405 |
|  |  |  |  |  |  |
| **id_GTA0017684** | 1 |  | 0.811 | 0.984 | 0.968 |
|  | 2 |  | 0.189 | 0.016 | 0.032 |
|  |  |  |  |  |  |
| **id_GTA0017692** | 1 |  | 0.003 | 0.011 | 0.671 |
|  | 2 |  | 0.997 | 0.989 | 0.329 |
|  |  |  |  |  |  |
| **id_GTA0017700** | 1 |  | 0.053 | 0.174 | 0.839 |
|  | 2 |  | 0.947 | 0.826 | 0.161 |
|  |  |  |  |  |  |
| **id_GTA0001788** | 1 |  | 0.161 | 0.455 | 0.911 |
|  | 2 |  | 0.839 | 0.545 | 0.089 |
|  |  |  |  |  |  |
| **id_GTA0017671** | 1 |  | 0.025 | 0.005 | 0.467 |
|  | 2 |  | 0.975 | 0.995 | 0.533 |
|  |  |  |  |  |  |
| **id_GTA0018195** | 1 |  | 0.176 | 0.053 | 0.696 |
|  | 2 |  | 0.824 | 0.947 | 0.304 |
|  |  |  |  |  |  |
| **id_GTA0017657** | 1 |  | 0.018 | 0.016 | 0.805 |
|  | 2 |  | 0.982 | 0.984 | 0.195 |

Table S 5 **Microsatellite allele frequencies for wildcats and domestic cats sampled in Luxembourg and Germany for the three clusters as identified by Structure. Genotypes not clearly assigned to any of the three clusters (***q^(i)^***<0.75) were excluded from this list (*n=*24 and *n=*39 for the roadkill and the hair trap datasets, respectively). WC_west, wildcats sampled in Luxembourg and western Germany; WC_central, wildcats sampled in central Germany; DC, domestic cats. Alleles in bold were only found in one of the two subspecies in that sample dataset.**

|  |  |  | **Roadkills (*n*=536)** | | |  | **Hair traps (*n*=535)** | |  |
| --- | --- | --- | --- | --- | --- | --- | --- | --- | --- |
| **Locus** | **Allele** |  | **WC_west** | **WC_central** | **DC** |  | **WC_west** | **WC_central** | **DC** |
| **FCA_171** | ***107*** |  | - | - | **0.009** |  | - | - | **0.011** |
|  | ***109*** |  | 0.005 | - | 0.118 |  | - | - | **0.080** |
|  | ***111*** |  | 0.088 | 0.097 | 0.325 |  | 0.082 | 0.143 | 0.351 |
|  | ***113*** |  | 0.104 | 0.007 | 0.392 |  | 0.133 | 0.005 | 0.402 |
|  | ***115*** |  | 0.279 | 0.570 | 0.108 |  | 0.390 | 0.557 | 0.092 |
|  | ***117*** |  | 0.449 | 0.319 | 0.042 |  | 0.379 | 0.283 | 0.040 |
|  | ***119*** |  | 0.061 | 0.007 | 0.005 |  | 0.017 | 0.009 | 0.023 |
|  | ***123*** |  | - | - | - |  | - | **0.005** | - |
|  | ***125*** |  | **0.005** | - | - |  | - | - | - |
|  | ***127*** |  | **0.003** | - | - |  | - | - | - |
|  | ***129*** |  | **0.005** | - | - |  | - | - | - |
|  |  |  |  |  |  |  |  |  |  |
| **FCA_8** | ***113*** |  | - | - | - |  | - | **0.002** | - |
|  | ***115*** |  | 0.131 | 0.114 | 0.008 |  | 0.039 | 0.124 | 0.011 |
|  | ***117*** |  | 0.003 | - | 0.125 |  | 0.006 | - | 0.133 |
|  | ***119*** |  | - | - | **0.004** |  | - | - | - |
|  | ***121*** |  | - | - | **0.004** |  | **0.003** | - | - |
|  | ***123*** |  | **0.008** | **0.116** | - |  | - | 0.073 | 0.006 |
|  | ***125*** |  | - | 0.003 | 0.004 |  | - | - | - |
|  | ***127*** |  | 0.005 | 0.019 | 0.020 |  | 0.020 | 0.013 | 0.006 |
|  | ***129*** |  | - | - | **0.157** |  | - | 0.004 | 0.228 |
|  | ***131*** |  | 0.131 | 0.312 | 0.173 |  | 0.156 | 0.367 | 0.089 |
|  | ***133*** |  | 0.306 | 0.212 | 0.093 |  | 0.330 | 0.237 | 0.106 |
|  | ***135*** |  | 0.230 | 0.024 | 0.230 |  | 0.221 | 0.018 | 0.150 |
|  | ***137*** |  | 0.101 | 0.003 | 0.089 |  | 0.126 | 0.009 | 0.156 |
|  | ***139*** |  | 0.038 | 0.198 | 0.052 |  | 0.070 | 0.135 | 0.078 |
|  | ***141*** |  | 0.043 | - | 0.028 |  | 0.031 | 0.018 | 0.033 |
|  | ***143*** |  | 0.005 | - | 0.012 |  | - | - | **0.006** |
|  |  |  |  |  |  |  |  |  |  |
| **FCA_571** | ***99*** |  | **0.005** | - | - |  | **0.003** | - | - |
|  | ***101*** |  | - | - | **0.036** |  | 0.006 | - | 0.022 |
|  | ***103*** |  | 0.136 | 0.103 | 0.085 |  | 0.153 | 0.056 | 0.132 |
|  | ***105*** |  | 0.229 | 0.024 | 0.052 |  | 0.124 | 0.065 | 0.093 |
|  | ***107*** |  | 0.151 | 0.156 | 0.383 |  | 0.158 | 0.125 | 0.368 |
|  | ***108*** |  | 0.274 | 0.222 | 0.319 |  | 0.316 | 0.185 | 0.258 |
|  | ***110*** |  | 0.033 | 0.011 | 0.101 |  | 0.023 | 0.004 | 0.088 |
|  | ***112*** |  | 0.075 | 0.458 | 0.020 |  | 0.085 | 0.496 | 0.027 |
|  | ***114*** |  | 0.030 | - | 0.004 |  | 0.065 | - | 0.005 |
|  | ***116*** |  | **0.008** | - | - |  | 0.011 | 0.002 | 0.005 |
|  | ***118*** |  | **0.003** | **0.003** | - |  | - | **0.016** | - |
|  | ***120*** |  | **0.058** | **0.024** | - |  | **0.054** | **0.049** | - |
|  | ***122*** |  | - | - | - |  | - | **0.002** | - |
|  | ***124*** |  | - | - | - |  | **0.003** | - | - |
|  |  |  |  |  |  |  |  |  |  |
| **FCA_124** | ***109*** |  |  |  |  |  | - | - | **0.005** |
|  | ***116*** |  | 0.089 | 0.008 | 0.099 |  | 0.051 | - | 0.082 |
|  | ***118*** |  | 0.077 | - | 0.116 |  | 0.129 | - | 0.082 |
|  | ***120*** |  | 0.048 | 0.106 | 0.417 |  | 0.059 | 0.119 | 0.401 |
|  | ***121*** |  | 0.041 | - | 0.004 |  | 0.042 | - | 0.022 |
|  | ***123*** |  | 0.020 | - | 0.004 |  | 0.022 | - | 0.033 |
|  | ***125*** |  | 0.015 | - | 0.037 |  | 0.045 | - | 0.049 |
|  | ***127*** |  | 0.497 | 0.193 | 0.070 |  | 0.466 | 0.175 | 0.038 |
|  | ***129*** |  | 0.117 | 0.653 | 0.198 |  | 0.140 | 0.639 | 0.214 |
|  | ***131*** |  | - | - | **0.054** |  | 0.022 | - | 0.071 |
|  | ***133*** |  | **0.092** | **0.040** | - |  | **0.022** | **0.066** | - |
|  | ***135*** |  | **0.003** | - | - |  |  |  |  |
|  |  |  |  |  |  |  |  |  |  |
| **FCA_149** | ***118*** |  | - | - | **0.041** |  | - | - | **0.044** |
|  | ***120*** |  | - | 0.003 | 0.124 |  | - | 0.002 | 0.093 |
|  | ***122*** |  | - | - | - |  | 0.006 | - | 0.005 |
|  | ***125*** |  | 0.207 | 0.024 | 0.355 |  | 0.235 | 0.031 | 0.275 |
|  | ***127*** |  | 0.464 | 0.455 | 0.174 |  | 0.464 | 0.458 | 0.198 |
|  | ***129*** |  | 0.270 | 0.495 | 0.231 |  | 0.243 | 0.469 | 0.330 |
|  | ***131*** |  | 0.059 | 0.024 | 0.074 |  | 0.053 | 0.040 | 0.044 |
|  | ***133*** |  | - | - | - |  | - | - | **0.011** |
|  |  |  |  |  |  |  |  |  |  |
| **FCA_275** | ***112*** |  | 0.418 | 0.310 | 0.179 |  | 0.389 | 0.392 | 0.157 |
|  | ***120*** |  | - | - | **0.022** |  | - | - | **0.045** |
|  | ***127*** |  | - | - | **0.004** |  | - | - | **-** |
|  | ***129*** |  | - | - | **0.049** |  | - | - | **0.039** |
|  | ***131*** |  | - | - | **0.013** |  | - | - | **0.017** |
|  | ***133*** |  | 0.120 | 0.036 | 0.250 |  | 0.105 | 0.043 | 0.230 |
|  | ***135*** |  | 0.182 | 0.082 | 0.116 |  | 0.325 | 0.070 | 0.197 |
|  | ***137*** |  | 0.171 | 0.563 | 0.054 |  | 0.078 | 0.489 | 0.084 |
|  | ***139*** |  | 0.109 | 0.003 | 0.165 |  | 0.096 | 0.005 | 0.135 |
|  | ***141*** |  | - | 0.005 | 0.094 |  | 0.006 | 0.002 | 0.073 |
|  | ***143*** |  | - | - | **0.018** |  | - | - | - |
|  | ***145*** |  | - | - | **0.036** |  | - | - | **0.022** |
|  |  |  |  |  |  |  |  |  |  |
| **FCA_170** | ***90*** |  | - | 0.005 | 0.169 |  | 0.006 | - | 0.297 |
|  | ***92*** |  | - | - | **0.008** |  | - | - | - |
|  | ***97*** |  | 0.040 | 0.111 | 0.012 |  | **0.053** | **0.146** | - |
|  | ***99*** |  | 0.078 | - | 0.048 |  | 0.092 | 0.002 | 0.049 |
|  | ***101*** |  | **0.010** | - | - |  | 0.014 | - | 0.005 |
|  | ***103*** |  | - | - | **0.008** |  | - | - | - |
|  | ***105*** |  | 0.058 | - | 0.069 |  | 0.073 | - | 0.066 |
|  | ***107*** |  | 0.207 | 0.042 | 0.125 |  | 0.078 | 0.035 | 0.099 |
|  | ***109*** |  | 0.225 | 0.148 | 0.157 |  | 0.249 | 0.122 | 0.088 |
|  | ***111*** |  | 0.318 | 0.272 | 0.056 |  | 0.358 | 0.288 | 0.055 |
|  | ***114*** |  | 0.010 | 0.296 | 0.024 |  | 0.003 | 0.288 | 0.060 |
|  | ***116*** |  | - | 0.056 | 0.056 |  | - | 0.073 | 0.044 |
|  | ***118*** |  | - | 0.008 | 0.085 |  | - | 0.002 | 0.099 |
|  | ***120*** |  | 0.003 | - | 0.109 |  | 0.003 | - | 0.082 |
|  | ***122*** |  | - | - | **0.036** |  | 0.028 | - | 0.027 |
|  | ***124*** |  | - | - | **0.024** |  | - | 0.004 | 0.011 |
|  | ***127*** |  | - | 0.003 | 0.008 |  | 0.006 | - | 0.005 |
|  | ***129*** |  | 0.005 | 0.003 | 0.004 |  | **0.003** | **0.002** | - |
|  | ***131*** |  | **0.045** | **0.053** | - |  | **0.036** | **0.038** | - |
|  | ***133*** |  | - | **0.003** | - |  | - | - | **0.005** |
|  | ***135*** |  | - | - | - |  | - | - | **0.005** |
|  |  |  |  |  |  |  |  |  |  |
| **FCA_88** | ***103*** |  | - | - | **0.004** |  | - | - | - |
|  | ***107*** |  | - | - | - |  | - | - | **0.011** |
|  | ***109*** |  | **0.003** | - | - |  | - | - | - |
|  | ***111*** |  | 0.008 | - | 0.008 |  | - | 0.002 | 0.033 |
|  | ***113*** |  | 0.008 | - | 0.049 |  | 0.008 | - | 0.077 |
|  | ***115*** |  | 0.169 | 0.246 | 0.049 |  | 0.165 | 0.239 | 0.055 |
|  | ***117*** |  | 0.386 | 0.720 | 0.061 |  | 0.374 | 0.706 | 0.071 |
|  | ***119*** |  | 0.081 | 0.003 | 0.107 |  | 0.159 | - | 0.071 |
|  | ***121*** |  | 0.227 | 0.032 | 0.459 |  | 0.134 | 0.049 | 0.401 |
|  | ***123*** |  | 0.119 | - | 0.094 |  | 0.148 | 0.004 | 0.126 |
|  | ***125*** |  | - | - | **0.041** |  | - | - | **0.055** |
|  | ***127*** |  | - | - | **0.107** |  | 0.011 | - | 0.082 |
|  | ***129*** |  | - | - | **0.020** |  | - | - | **0.016** |
|  |  |  |  |  |  |  |  |  |  |
| **FCA_364** | ***104*** |  | - | - | **0.024** |  | - | - | **0.018** |
|  | ***113*** |  | 0.008 | - | 0.137 |  | 0.049 | - | 0.116 |
|  | ***114*** |  | - | - | **0.008** |  | - | - | **0.018** |
|  | ***116*** |  | 0.030 | 0.005 | 0.169 |  | 0.061 | 0.005 | 0.226 |
|  | ***118*** |  | 0.294 | 0.459 | 0.141 |  | 0.220 | 0.484 | 0.067 |
|  | ***120*** |  | 0.055 | 0.038 | 0.250 |  | 0.084 | 0.068 | 0.329 |
|  | ***122*** |  | 0.400 | 0.468 | 0.210 |  | 0.390 | 0.416 | 0.177 |
|  | ***124*** |  | 0.214 | 0.030 | 0.056 |  | 0.197 | 0.027 | 0.043 |
|  | ***126*** |  | - | - | **0.004** |  | - | - | **0.006** |
|  |  |  |  |  |  |  |  |  |  |
| **FCA_576** | ***131*** |  | **0.060** | - | - |  | 0.053 | - | 0.016 |
|  | ***133*** |  | 0.128 | 0.212 | 0.012 |  | 0.073 | 0.201 | 0.005 |
|  | ***135*** |  | 0.003 | - | 0.085 |  | 0.008 | 0.002 | 0.044 |
|  | ***138*** |  | 0.003 | - | 0.008 |  | 0.003 | - | 0.016 |
|  | ***140*** |  | 0.010 | - | 0.008 |  | - | 0.002 | 0.022 |
|  | ***142*** |  | 0.018 | 0.077 | 0.121 |  | 0.031 | 0.044 | 0.126 |
|  | ***145*** |  | - | - | **0.149** |  | - | - | **0.115** |
|  | ***147*** |  | 0.538 | 0.593 | 0.480 |  | 0.548 | 0.628 | 0.522 |
|  | ***149*** |  | 0.236 | 0.119 | 0.056 |  | 0.250 | 0.122 | 0.027 |
|  | ***151*** |  | 0.005 | - | 0.077 |  | 0.034 | - | 0.099 |
|  | ***153*** |  | - | - | **0.004** |  | - | - | **0.005** |
|  |  |  |  |  |  |  |  |  |  |
| **FCA_132** | ***136*** |  | - | 0.003 | 0.228 |  | - | 0.002 | 0.154 |
|  | ***138*** |  | - | - | **0.041** |  | 0.006 | - | 0.093 |
|  | ***140*** |  | - | - | **0.138** |  | - | 0.002 | 0.104 |
|  | ***142*** |  | - | - | **0.008** |  | - | - | - |
|  | ***144*** |  | 0.005 | - | 0.130 |  | 0.020 | - | 0.126 |
|  | ***145*** |  | **0.085** | - | - |  | **0.067** | **0.002** | - |
|  | ***148*** |  | 0.049 | 0.003 | 0.016 |  | 0.053 | 0.002 | 0.011 |
|  | ***150*** |  | 0.023 | 0.128 | 0.069 |  | 0.039 | 0.082 | 0.093 |
|  | ***152*** |  | 0.103 | 0.306 | 0.126 |  | 0.129 | 0.310 | 0.088 |
|  | ***154*** |  | 0.223 | 0.024 | 0.106 |  | 0.138 | 0.049 | 0.132 |
|  | ***156*** |  | 0.292 | 0.335 | 0.061 |  | 0.306 | 0.279 | 0.060 |
|  | ***158*** |  | 0.187 | 0.011 | 0.061 |  | 0.185 | 0.040 | 0.110 |
|  | ***160*** |  | 0.005 | - | 0.008 |  | 0.008 | - | 0.022 |
|  | ***162*** |  | **0.028** | **0.191** | - |  | 0.048 | 0.232 | 0.005 |
|  | ***164*** |  | - | - | **0.004** |  | - | - | - |
|  | ***166*** |  | - | - | **0.004** |  | - | - | - |
|  |  |  |  |  |  |  |  |  |  |
| **FCA_567** | ***90*** |  | - | - | **0.004** |  | - | - | - |
|  | ***92*** |  | 0.018 | - | 0.074 |  | 0.062 | - | 0.077 |
|  | ***94*** |  | **0.005** | - | - |  | 0.008 | - | 0.022 |
|  | ***96*** |  | 0.128 | 0.168 | 0.061 |  | 0.149 | 0.180 | 0.060 |
|  | ***98*** |  | 0.217 | 0.381 | 0.189 |  | 0.191 | 0.329 | 0.176 |
|  | ***100*** |  | 0.145 | 0.054 | 0.029 |  | 0.154 | 0.049 | 0.044 |
|  | ***102*** |  | 0.153 | 0.008 | 0.410 |  | 0.112 | 0.004 | 0.368 |
|  | ***104*** |  | 0.253 | 0.311 | 0.230 |  | 0.199 | 0.331 | 0.225 |
|  | ***106*** |  | 0.074 | 0.078 | 0.004 |  | 0.110 | 0.104 | 0.016 |
|  | ***108*** |  | **0.008** | - | - |  | 0.014 | 0.002 | 0.011 |
|  |  |  |  |  |  |  |  |  |  |
| **FCA_347** | ***158*** |  | 0.378 | 0.212 | 0.148 |  | 0.383 | 0.218 | 0.205 |
|  | ***160*** |  | 0.024 | 0.008 | 0.016 |  | 0.059 | 0.020 | 0.011 |
|  | ***162*** |  | **0.035** | **0.042** | - |  | 0.067 | 0.053 | 0.011 |
|  | ***164*** |  | 0.351 | 0.579 | 0.148 |  | 0.338 | 0.549 | 0.131 |
|  | ***166*** |  | 0.013 | 0.013 | 0.201 |  | 0.017 | 0.016 | 0.205 |
|  | ***168*** |  | - | - | **0.004** |  | - | - | **0.006** |
|  | ***170*** |  | 0.003 | 0.003 | 0.078 |  | - | - | **0.074** |
|  | ***172*** |  | 0.040 | - | 0.221 |  | 0.056 | - | 0.210 |
|  | ***174*** |  | - | - | **0.041** |  | - | - | **0.045** |
|  | ***176*** |  | 0.003 | - | 0.025 |  | - | - | **0.023** |
|  | ***178*** |  | 0.154 | 0.143 | 0.102 |  | 0.078 | 0.144 | 0.057 |
|  | ***180*** |  | - | - | **0.016** |  | 0.003 | - | 0.023 |
|  |  |  |  |  |  |  |  |  |  |
| **FCA_232** | ***95*** |  | 0.023 | - | 0.149 |  | 0.067 | - | 0.203 |
|  | ***97*** |  | 0.005 | - | 0.141 |  | 0.011 | 0.002 | 0.165 |
|  | ***99*** |  | 0.008 | - | 0.004 |  | - | **0.002** | - |
|  | ***102*** |  | 0.013 | - | 0.016 |  | **0.017** | **0.004** | - |
|  | ***104*** |  | 0.437 | 0.382 | 0.113 |  | 0.483 | 0.336 | 0.121 |
|  | ***106*** |  | 0.129 | 0.155 | 0.040 |  | 0.121 | 0.184 | 0.082 |
|  | ***108*** |  | 0.336 | 0.455 | 0.153 |  | 0.244 | 0.465 | 0.110 |
|  | ***110*** |  | 0.008 | - | 0.040 |  | 0.011 | - | 0.044 |
|  | ***112*** |  | 0.033 | - | 0.113 |  | 0.034 | - | 0.088 |
|  | ***114*** |  | 0.003 | - | 0.065 |  | 0.003 | - | 0.055 |
|  | ***116*** |  | 0.008 | 0.008 | 0.161 |  | 0.008 | 0.007 | 0.126 |
|  | ***118*** |  | - | - | **0.004** |  | - | - | **0.005** |
|  |  |  |  |  |  |  |  |  |  |

Table S 6 Values represent absolute numbers of assignment of individuals using **Structure** and **NewHybrids.** All hybrid categories in **NewHybrids** are summed up to one hybrid group. For the hair trap dataset 19 individuals which could not be assigned to any of the west or central wildcat clusters (*q^(i)^*<0.75) in the **STRUCTURE** analyses were excluded; also 19 individuals which were analyzed in both the western and central dataset, which were sampled in the area of overlap of the western and central clusters, were excluded.

|  |  |  | Structure | | |
| --- | --- | --- | --- | --- | --- |
|  | NewHybrids |  | **WC** | **DC** | **admixed** |
| hair trap dataset, msats | **WC** |  | 400 | - | - |
|  | **DC** |  | - | 45 | - |
|  | **hybrids** |  | - | 4 | 4 |
|  | **not assigned** |  | 5 | 23 | 16 |
| roadkill dataset, msats | **WC** |  | 386 | - | 2 |
|  | **DC** |  | - | 121 | 2 |
|  | **hybrids** |  | - | - | - |
|  | **not assigned** |  | 2 | 3 | 11 |
| roadkill dataset, SNPs | **WC** |  | 401 | - | - |
|  | **DC** |  | - | 122 | - |
|  | **hybrids** |  | 1 | - | 12 |
|  | **not assigned** |  | - | - | - |

Table S 7 Comparison of parental or hybrid categories for the roadkill dataset using **NewHybrids**. Disagreements are shown as percentage of individuals with differing classifications. Individuals that could not be assigned to any of the three clusters (wc_west, wc_central, domestic cats) were excluded (*n*=9). Five individuals indicated within brackets were collected in the area of overlap of the western and central clusters and were, therefore, run in both microsatellite **NewHybrids** runs but could only be assigned to a category with *q^(i)^*<0.85 in one run.

|  | **SNPs** | | | | | |
| --- | --- | --- | --- | --- | --- | --- |
|  | | | | | |
| **Msats** | **WC** | **DC** | **F1** | **F2** | **BC_WC** | **BC_DC** |
|  |  |  |  |  |  |  |
| **WC** | 383 (1) | - | - | - | 4 | - |
| **DC** | - | 115 (3) | 1 | - | 1 (1) | 2 |
| **F1** | - | - | - | - | - | - |
| **F2** | - | - | - | - | - | - |
| **BC_WC** | - | - | - | - | - | - |
| **BC_DC** | - | - | - | - | - | - |
| **Assigned with *q^(i)^*<0.85** | 8 | 4 | 1 | - | 2 | 1 |

Table S 8 Assignment of haplotypes to wildcat or domestic cat based on **NewHybrids** analysis. Individuals that were not assigned to any of the west or central wildcat clusters (*q^(i)^*<0.75) in the **Structure** analyses were excluded from the **NewHybrids** analyses (9 individuals from the roadkill dataset).

| haplotype | SNP dataset (*n*=536) | | | | | |  |  | Msat dataset (*n*=527) | | |
| --- | --- | --- | --- | --- | --- | --- | --- | --- | --- | --- | --- |
| **SNG-HP-FS** | **WC** | **DC** | **F1** | **BC_WC** | **BC_DC** | ***n*** | ***classification*** |  | **WC** | **DC** | **unassigned** |
| **03** | 110 | 0 | 0 | 1 | 0 | 111 | wildcat |  | 104 | 0 | 4 |
| **04** | 84 | 0 | 0 | 4 | 1 | 89 | wildcat |  | 80 | 1 | 7 |
| **05** | 25 | 0 | 0 | 1 | 0 | 26 | wildcat |  | 25 | 1 | 0 |
| **06** | 46 | 0 | 0 | 1 | 0 | 47 | wildcat |  | 47 | 0 | 0 |
| **07** | 4 | 0 | 0 | 0 | 0 | 4 | - |  | 4 | 0 | 0 |
| **13** | 1 | 3 | 0 | 0 | 0 | 4 | - |  | 1 | 2 | 1 |
| **15** | 0 | 1 | 0 | 0 | 0 | 1 | - |  | 0 | 1 | 0 |
| **16** | 0 | 73 | 0 | 0 | 1 | 74 | domestic cat |  | 0 | 70 | 4 |
| **18** | 0 | 1 | 0 | 0 | 0 | 1 | - |  | 0 | 1 | 0 |
| **22** | 124 | 0 | 1 | 1 | 1 | 127 | wildcat |  | 119 | 0 | 3 |
| **23** | 1 | 0 | 0 | 0 | 0 | 1 | - |  | 1 | 0 | 0 |
| **26** | 0 | 3 | 0 | 0 | 0 | 3 | - |  | 0 | 3 | 0 |
| **32** | 0 | 20 | 0 | 0 | 0 | 20 | domestic cat |  | 0 | 19 | 1 |
| **34** | 0 | 7 | 0 | 0 | 0 | 7 | - |  | 0 | 7 | 0 |
| **36** | 0 | 5 | 0 | 0 | 0 | 5 | - |  | 0 | 4 | 1 |
| **37** | 0 | 4 | 0 | 0 | 0 | 4 | - |  | 0 | 4 | 0 |
| **39** | 0 | 2 | 0 | 0 | 0 | 2 | - |  | 0 | 2 | 0 |
| **40** | 5 | 0 | 1 | 0 | 0 | 6 | - |  | 5 | 1 | 0 |
| **49** | 0 | 1 | 0 | 0 | 0 | 1 | - |  | 0 | 1 | 0 |
| **52** | 0 | 1 | 0 | 0 | 0 | 1 | - |  | 0 | 1 | 0 |
| **54** | 1 | 0 | 0 | 0 | 0 | 1 | - |  | 1 | 0 | 0 |
| **62** | 0 | 1 | 0 | 0 | 0 | 1 | - |  | 0 | 1 | 0 |

#### Table S 9 Migration rates assessed using the software BayesAss (Wilson and Rannala 2003). Shown values are the number of individuals of the first named group that derived from the other group per generation. Standard deviation of marginal posterior distribution is shown in brackets.

| **marker type** | **parental group** | **migration rate** |
| --- | --- | --- |
| Msats  central | **WC - DC** | 0.0018 (0.0018) |
|  | **DC - WC** | 0.0040 (0.0040) |
| Msats  western | **WC -DC** | 0.0018 (0.0018) |
|  | **DC -WC** | 0.0046 (0.0045) |
| SNPs  central | **WC -DC** | 0.0029 (0.0024) |
|  | **DC -WC** | 0.0040 (0.0040) |
| SNPs western | **WC – DC** | 0.0050 (0.0029) |
|  | **DC - WC** | 0.0131 (0.0074) |

Table S 10 Genotypes generated using **Hybridlab** and assigned with **Structure**. The numbers are percentage of individuals (*n*=200) assigned to the respective cluster.

|  |  |  |  | | assigned category with Structure | | | |
| --- | --- | --- | --- | --- | --- | --- | --- | --- |
|  | **simulated**  **hybrid**  **category** |  | **cluster 1 (%)** |  | | **cluster 2 (%)** |  | **not classified (*q^(i)^*<0.75; %)** |
| Msats  central | **WC** |  | 100 |  | | 0 |  | 0 |
|  | **DC** |  | 0 |  | | 98 |  | 2 |
|  | **F1** |  | 2 |  | | 1 |  | 98 |
|  | **F2** |  | 5 |  | | 5 |  | 91 |
|  | **BC_WC** |  | 48 |  | | 0 |  | 52 |
|  | **BC_DC** |  | 0 |  | | 50 |  | 50 |
|  | **BC_wc_wc** |  | 89 |  | | 0 |  | 12 |
|  | **BC_dc_dc** |  | 0 |  | | 80 |  | 20 |
| Msats  western | **WC** |  | 100 |  | | 0 |  | 1 |
|  | **DC** |  | 0 |  | | 97 |  | 3 |
|  | **F1** |  | 4 |  | | 5 |  | 92 |
|  | **F2** |  | 5 |  | | 5 |  | 90 |
|  | **BC_WC** |  | 54 |  | | 0 |  | 47 |
|  | **BC_DC** |  | 0 |  | | 46 |  | 54 |
|  | **BC_wc_wc** |  | 86 |  | | 0 |  | 14 |
|  | **BC_dc_dc** |  | 0 |  | | 84 |  | 16 |
| SNPs | **WC** |  | 100 |  | | 0 |  | 0 |
|  | **DC** |  | 0 |  | | 100 |  | 0 |
|  | **F1** |  | 0 |  | | 0 |  | 100 |
|  | **F2** |  | 0 |  | | 0 |  | 100 |
|  | **BC_WC** |  | 40 |  | | 0 |  | 60 |
|  | **BC_DC** |  | 0 |  | | 37 |  | 63 |
|  | **BC_wc_wc** |  | 100 |  | | 0 |  | 1 |
|  | **BC_dc_dc** |  | 0 |  | | 99 |  | 1 |

Table S 11 Mean *q^(i)^*-values for **Structure** and ranges of simulated genotypes for their respective hybrid category are shown. Rates of not classified genotypes are reported. *Q^(i)^*-values above 0.75 are represented as clearly assigned.

| **simulated**  **genotypes**  **for:** | **simulated**  **Hybrid**  **category** |  | **mean *q^(i)^*-value (range)**  **Structure** |  | **not classified**  **(*q^(i)^*<0.75; %)** |
| --- | --- | --- | --- | --- | --- |
| Msats  central | **WC** |  | 0.95 (0.859-0.971) |  | 0 |
|  | **DC** |  | 0.082 (0.026-0.38) |  | 2 |
|  | **F1** |  | 0.503 (0.219-0.782) |  | 98 |
|  | **F2** |  | 0.501 (0.15-0.894) |  | 91 |
|  | **BC_WC** |  | 0.738 (0.48-0.967) |  | 52 |
|  | **BC_DC** |  | 0.261 (0.041-0.575) |  | 50 |
|  | **BC_wc_wc** |  | 0.852 (0.485-0.972) |  | 12 |
|  | **BC_dc_dc** |  | 0.167 (0.044-0.508) |  | 20 |
| Msats  western | **WC** |  | 0.927 (0.741-0.968) |  | 1 |
|  | **DC** |  | 0.098 (0.034-0.373) |  | 3 |
|  | **F1** |  | 0.5 (0.173-0.846) |  | 92 |
|  | **F2** |  | 0.493 (0.128-0.904) |  | 90 |
|  | **BC_WC** |  | 0.756 (0.367-0.952) |  | 47 |
|  | **BC_DC** |  | 0.275 (0.044-0.73) |  | 54 |
|  | **BC_wc_wc** |  | 0.844 (0.361-0.965) |  | 14 |
|  | **BC_dc_dc** |  | 0.161 (0.036-0.655) |  | 16 |
| SNPs | **WC** |  | 0.977 (0.921-0.993) |  | 0 |
|  | **DC** |  | 0.035 (0.009-0.129) |  | 0 |
|  | **F1** |  | 0.505 (0.407-0.586) |  | 100 |
|  | **F2** |  | 0.501 (0.279-0.631) |  | 100 |
|  | **BC_WC** |  | 0.74 (0.609-0.85) |  | 60 |
|  | **BC_DC** |  | 0.266 (0.159-0.406) |  | 63 |
|  | **BC_wc_wc** |  | 0.859 (0.734-0.949) |  | 1 |
|  | **BC_dc_dc** |  | 0.148 (0.06-0.281) |  | 1 |

Table S 12 Genotypes generated with **Hybridlab** and assigned with **NewHybrids**. The numbers are percentage of individuals (*n*=200) assigned to the respective cluster.

|  |  |  | assigned category with NewHybrids | | | | | |  |  |
| --- | --- | --- | --- | --- | --- | --- | --- | --- | --- | --- |
|  | **simulated**  **hybrid**  **category** |  | **WC** | **DC** | **F1** | **F2** | **BC_WC** | **BC_DC** |  | **not**  **clearly**  **assigned (*q^(i)^*<0.85)** |
| Msats  central | **WC** |  | 99 | 0 | 0 | 0 | 0 | 0 |  | 1 |
|  | **DC** |  | 0 | 79 | 0 | 1 | 0 | 0 |  | 21 |
|  | **F1** |  | 0 | 0 | 32 | 0 | 0 | 0 |  | 68 |
|  | **F2** |  | 0 | 0 | 2 | 2 | 8 | 0 |  | 88 |
|  | **BC_WC** |  | 8 | 0 | 0 | 0 | 20 | 0 |  | 72 |
|  | **BC_DC** |  | 0 | 2 | 0 | 0 | 0 | 6 |  | 92 |
|  | **BC_wc_wc** |  | 38 | 0 | 0 | 0 | 8 | 0 |  | 54 |
|  | **BC_dc_dc** |  | 0 | 34 | 0 | 0 | 0 | 2 |  | 64 |
| Msats  western | **WC** |  | 96 | 0 | 0 | 0 | 0 | 0 |  | 5 |
|  | **DC** |  | 0 | 83 | 0 | 0 | 0 | 0 |  | 18 |
|  | **F1** |  | 0 | 0 | 0 | 0 | 0 | 0 |  | 100 |
|  | **F2** |  | 2 | 0 | 0 | 0 | 0 | 0 |  | 98 |
|  | **BC_WC** |  | 28 | 0 | 0 | 2 | 0 | 0 |  | 70 |
|  | **BC_DC** |  | 0 | 18 | 2 | 0 | 0 | 0 |  | 80 |
|  | **BC_wc_wc** |  | 78 | 0 | 0 | 0 | 0 | 0 |  | 22 |
|  | **BC_dc_dc** |  | 0 | 46 | 0 | 0 | 0 | 0 |  | 54 |
| SNPs | **WC** |  | 100 | 0 | 0 | 0 | 0 | 0 |  | 0 |
|  | **DC** |  | 0 | 100 | 0 | 0 | 0 | 0 |  | 0 |
|  | **F1** |  | 0 | 0 | 100 | 0 | 0 | 0 |  | 0 |
|  | **F2** |  | 0 | 0 | 0 | 96 | 0 | 2 |  | 2 |
|  | **BC_WC** |  | 0 | 0 | 0 | 2 | 96 | 0 |  | 2 |
|  | **BC_DC** |  | 0 | 0 | 0 | 0 | 0 | 98 |  | 2 |
|  | **BC_wc_wc** |  | 12 | 0 | 0 | 0 | 78 | 0 |  | 10 |
|  | **BC_dc_dc** |  | 0 | 40 | 0 | 0 | 0 | 44 |  | 16 |

Table S 13 Mean *q^(i)^* values and ranges of simulated genotypes for their respective hybrid category for the **NewHybrids** results. *Q^(i)^*-values above 0.85 was used as a threshold with our empirical data set.

| **simulated**  **genotypes**  **for:** | **simulated**  **hybrid**  **category** | **mean *q^(i)^*-value (range)**  **NewHybrids** | **classified**  **to wrong**  **category (%)** | **assigned with**  ***q^(i)^*<0.85 (%)** |
| --- | --- | --- | --- | --- |
| Msats  central | **WC** | 0.987 (0.792- 0.999) | 0 | 1 |
|  | **DC** | 0.895 (0.051 - 1) | 1 | 21 |
|  | **F1** | 0.653 (0.007 - 0.973) | 0 | 68 |
|  | **F2** | 0.362 (0.014 - 0.862) | 10 | 88 |
|  | **BC_WC** | 0.614 (0.006 - 0.947) | 8 | 72 |
|  | **BC_DC** | 0.514 (0.007 - 0.915) | 2 | 92 |
| Msats  western | **WC** | 0.971 (0.535 - 0.999) | 0 | 5 |
|  | **DC** | 0.909 (0.159 - 0.999) | 0 | 18 |
|  | **F1** | 0.437 (0.055 - 0.847) | 0 | 100 |
|  | **F2** | 0.414 (0.007 - 0.844) | 2 | 98 |
|  | **BC_WC** | 0.207 (0.004 - 0.597) | 30 | 70 |
|  | **BC_DC** | 0.228 (0.028 - 0.570) | 20 | 80 |
| SNPs | **WC** | 0.999(0.999 - 1) | 0 | 0 |
|  | **DC** | 0.999 (0.984 - 1) | 0 | 0 |
|  | **F1** | 0.999 (0.999 - 1) | 0 | 0 |
|  | **F2** | 0.960(0.003 - 1) | 2 | 2 |
|  | **BC_WC** | 0.973 (0.055 - 1) | 2 | 2 |
|  | **BC_DC** | 0.991 (0.606 - 1) | 0 | 2 |

#### Table S 14 Samples with *q^(i)^*<0.85 in NewHybrids using microsatellite genotypes, showing their corresponding SNP and mtDNA results. Parental wildcat= WC; parental domestic cat= DC; first generation hybrid= F1; second generation hybrid= F2; backcross to parental wildcat= BC_WC; backcross to parental domestic cat= BC_DC; not run= sample was not analysed with SNPs; bad quality= SNP analyses could not be performed due to low number of amplified SNP loci.

| **dataset** |  | **Highest probability** | | | | **Second highest probability** | | | | **mtDNA assignment** | **SNPs** |
| --- | --- | --- | --- | --- | --- | --- | --- | --- | --- | --- | --- |
|  | **Individual ID** | **JJ** | **JU** | **UJ** | **UU** | **JJ** | **JU** | **UJ** | **UU** |  | **all P ≥ 0.85** |
| **roadkills west** | BY_04_M_02_O_0015_i01 | F2 | F2 | F2 | F2 | BC_WC | BC_WC | DC | DC | wildcat | BC_WC |
|  | HE_13_M_02_O_0004_i01 | F2 | F2 | DC | DC | BC_DC | BC_DC | F2 | F2 | domestic | DC |
|  | HE_16_F_02_O_0010_i01^X)^ | F2 | F2 | F2 | F2 | BC_WC | BC_WC | DC | BC_DC | domestic | DC |
|  | HE_16_M_01_O_0028_i01 | F2 | F2 | F2 | F2 | BC_DC | BC_DC | DC | BC_DC | domestic | DC |
|  | HE_16_M_06_O_1033_i01 | F2 | F2 | F2 | F2 | BC_WC | BC_WC | WC | WC | domestic | DC |
|  | LU_03_M_01_O_0022_i01 | F2 | F2 | F2 | F2 | BC_WC | BC_WC | WC | BC_WC | wildcat | WC |
|  | LU_04_M_03_O_0031_i01 | F2 | F2 | WC | WC | WC | WC | F2 | F2 | wildcat | BC_WC |
|  | RP_03_M_03_O_0027_i01 | BC_WC | BC_WC | WC | WC | F2 | F2 | F2 | BC_WC | wildcat | WC |
|  | RP_04_F_02_O_0112_i01 | WC | BC_WC | WC | WC | BC_WC | WC | F2 | BC_WC | wildcat | WC |
|  | RP_04_M_02_O_0132_i01 | BC_WC | BC_WC | WC | WC | WC | F2 | F2 | BC_WC | wildcat | WC |
|  | RP_04_M_03_O_0043_i01 | F2 | BC_WC | WC | WC | BC_WC | F2 | F2 | F2 | wildcat | WC |
|  | RP_16_M_03_O_0015_i01 | F2 | F2 | DC | DC | BC_DC | BC_DC | F2 | F2 | domestic | DC |
|  | RP_22_M_03_O_0024_i01 | F2 | BC_WC | F2 | F2 | BC_WC | F2 | WC | BC_WC | wildcat | F1 |
|  | RP_32_M_03_O_0031_i01 | F2 | F2 | F2 | F2 | BC_DC | BC_DC | DC | DC | domestic | DC |
| **roadkills central** | BY_03_M_02_O_0190_i01 | BC_WC | BC_WC | WC | WC | WC | WC | BC_WC | BC_WC | wildcat | WC |
|  | BY_04_M_05_O_0071_i01 | BC_WC | BC_WC | BC_WC | BC_WC | F2 | F1 | F2 | F1 | wildcat | WC |
|  | HE_03_F_02_O_0896_i01 | BC_WC | BC_WC | BC_WC | BC_WC | F2 | F2 | F2 | F2 | wildcat | BC_WC |
|  | HE_16_F_02_O_0010_i01^X)^ | DC | F2 | F2 | F2 | F2 | BC_DC | DC | BC_DC | domestic | DC |
|  | RP_22_M_02_O_0155_i01 | BC_WC | BC_WC | F2 | F2 | F2 | F2 | BC_WC | BC_WC | wildcat | WC |
|  | TH_04_M_02_B_0072_i01 | BC_WC | BC_WC | WC | WC | WC | F2 | BC_WC | BC_WC | wildcat | WC |
|  | TH_22_M_02_B_0071_i01 | BC_WC | BC_WC | BC_WC | BC_WC | F2 | F2 | F2 | F2 | wildcat | BC_DC |
|  | TH_36_M_02_O_0077_i01 | DC | DC | DC | DC | BC_DC | BC_DC | F2 | BC_DC | domestic | DC |
| **hair traps west** | HE_00_F_03_O_0098_i01 | BC_DC | BC_DC | BC_DC | BC_DC | F2 | F2 | DC | DC | domestic | not run |
|  | HE_00_M_03_O_0247_i01 | BC_DC | BC_DC | BC_DC | BC_DC | F2 | F2 | DC | DC | unassigned | DC |
|  | HE_03_F_18_B_0737_i05^X)^ | BC_WC | BC_WC | BC_WC | BC_WC | F2 | F2 | F2 | F2 | wildcat | BC_WC |
|  | HE_03_M_03_O_0791_i01^X)^ | BC_WC | BC_WC | WC | WC | F2 | F2 | BC_WC | BC_WC | wildcat | F1 |
|  | HE_16_M_03_B_0864_i01 | BC_DC | BC_DC | BC_DC | BC_DC | F2 | F2 | DC | DC | domestic | DC |
|  | HE_16_M_03_O_0237_i01 | F2 | F2 | BC_DC | BC_DC | BC_DC | BC_DC | DC | DC | domestic | DC |
|  | HE_16_M_03_O_0938_i01 | F2 | F2 | BC_DC | BC_DC | BC_DC | BC_DC | DC | DC | domestic | DC |
|  | HE_16_M_09_O_0471_i03 | F2 | F2 | BC_DC | BC_DC | BC_WC | BC_WC | F2 | F2 | domestic | DC |
|  | HE_22_M_15_O_1201_i05^X)^ | BC_WC | BC_WC | BC_WC | BC_WC | F2 | F2 | F2 | F2 | wildcat | bad quality |
|  | HE_23_M_09_O_1100_i03^X)^ | BC_WC | BC_WC | WC | WC | F2 | F2 | BC_WC | BC_WC | wildcat | F2 |
|  | HE_32_M_03_O_0263_i01 | BC_DC | BC_DC | BC_DC | BC_DC | F2 | F2 | DC | DC | domestic | DC |
|  | HE_34_F_03_O_0942_i01 | F2 | F2 | BC_DC | BC_DC | BC_DC | BC_DC | DC | F2 | domestic | DC |
|  | HE_34_M_03_O_0514_i01^X)^ | F2 | F2 | BC_DC | BC_DC | BC_WC | BC_WC | F2 | F2 | domestic | DC |
|  | HE_34_M_09_G_0479_i03 | F2 | F2 | BC_DC | BC_DC | BC_WC | BC_WC | F2 | F2 | domestic | DC |
|  | HE_48_M_03_O_0936_i01 | F2 | F2 | BC_DC | BC_DC | BC_DC | BC_DC | DC | DC | unassigned | DC |
|  | LU_16_F_03_C_0127_i01 | BC_WC | BC_WC | BC_WC | BC_WC | F2 | F2 | WC | WC | domestic | BC_WC |
|  | LU_16_F_03_G_0148_i01 | F2 | F2 | BC_DC | BC_DC | BC_WC | BC_WC | BC_WC | F2 | domestic | F1 |
|  | LU_16_F_09_O_0125_i03 | BC_WC | BC_WC | WC | WC | WC | WC | BC_WC | BC_WC | domestic | BC_WC |
|  | LU_16_M_18_B_0173_i06 | BC_WC | BC_WC | BC_WC | BC_WC | F2 | F2 | F2 | F2 | domestic | bad quality |
|  | LU_16_M_18_G_0159_i06 | WC | WC | WC | WC | BC_WC | BC_WC | BC_WC | BC_WC | domestic | BC_WC |
|  | LU_22_F_06_O_0055_i02 | F2 | F2 | BC_WC | F2 | BC_WC | BC_WC | BC_DC | BC_WC | wildcat | BC_WC |
|  | NW_00_F_03_O_0167_i01 | BC_DC | BC_DC | DC | DC | F2 | F2 | BC_DC | BC_DC | domestic | DC |
|  | NW_00_M_03_O_0166_i01 | BC_DC | BC_DC | DC | DC | F2 | F2 | BC_DC | BC_DC | domestic | DC |
|  | NW_00_M_06_O_0170_i02 | BC_DC | BC_DC | DC | DC | F2 | F2 | BC_DC | BC_DC | domestic | DC |
|  | NW_16_F_03_O_0122_i01 | BC_DC | BC_DC | BC_DC | BC_DC | F2 | F2 | DC | DC | domestic | DC |
|  | NW_16_M_03_G_0136_i01 | BC_DC | BC_DC | DC | DC | F2 | F2 | BC_DC | BC_DC | domestic | DC |
|  | NW_16_M_03_G_0191_i01 | F2 | BC_DC | BC_DC | BC_DC | BC_DC | F2 | DC | DC | domestic | DC |
|  | NW_16_M_03_O_0012_i01 | BC_DC | BC_DC | BC_DC | BC_DC | F2 | F2 | DC | DC | domestic | DC |
|  | NW_16_M_03_O_0035_i01 | BC_DC | BC_DC | DC | DC | F2 | F2 | BC_DC | BC_DC | domestic | DC |
|  | NW_16_M_03_O_0140_i01 | F2 | F2 | DC | DC | BC_DC | BC_DC | BC_DC | BC_DC | domestic | DC |
|  | NW_16_M_03_O_0159_i01 | F2 | F2 | BC_DC | BC_DC | BC_DC | BC_DC | DC | DC | domestic | DC |
|  | NW_16_M_06_G_0141_i02 | BC_DC | BC_DC | BC_DC | BC_DC | F2 | F2 | DC | DC | domestic | DC |
|  | NW_16_M_24_C_0126_i08 | BC_DC | BC_DC | DC | DC | F2 | F2 | BC_DC | BC_DC | domestic | DC |
|  | NW_36_M_03_O_0475_i01 | F2 | F2 | BC_DC | DC | BC_DC | BC_DC | DC | BC_DC | domestic | DC |
|  | RP_00_F_03_O_0115_i01 | BC_DC | BC_DC | BC_DC | BC_DC | F2 | F2 | DC | DC | domestic | BC_DC |
|  | RP_16_M_03_O_0251_i01 | F2 | F2 | BC_DC | BC_DC | BC_DC | BC_DC | DC | DC | domestic | DC |
|  | RP_22_F_03_G_0557_i01 | BC_WC | BC_WC | WC | WC | F2 | F2 | BC_WC | BC_WC | wildcat | WC |
|  | RP_22_M_15_B_0054_i05^X)^ | F2 | F2 | BC_DC | BC_DC | BC_WC | BC_WC | F2 | F2 | wildcat | not run |
|  | SL_00_F_03_O_0031_i01 | F2 | F2 | BC_DC | BC_DC | BC_DC | BC_DC | DC | DC | domestic | DC |
|  | SL_00_M_03_C_0029_i01 | BC_DC | BC_DC | DC | DC | F2 | F2 | BC_DC | BC_DC | unassigned | BC_DC |
|  | SL_00_M_03_O_0030_i01 | F2 | F2 | BC_DC | BC_DC | BC_DC | BC_DC | DC | DC | domestic | DC |
|  | SL_04_M_03_O_0078_i01 | BC_WC | BC_WC | BC_WC | BC_WC | F2 | F2 | WC | WC | wildcat | WC |
|  | SL_16_F_03_O_0018_i01 | F2 | F2 | BC_DC | BC_DC | BC_DC | BC_DC | DC | DC | domestic | DC |
|  | SL_16_M_03_O_0017_i01 | F2 | F2 | BC_DC | BC_DC | BC_DC | BC_DC | DC | DC | domestic | DC |
|  | SL_40_M_03_O_0036_i01 | F2 | F2 | BC_DC | BC_DC | BC_DC | BC_DC | DC | DC | wildcat | F1 |
| **hair traps central** | BY_00_F_06_O_0046_i02 | F2 | F2 | F2 | F2 | BC_WC | BC_WC | BC_WC | BC_WC | wildcat | not assigned |
|  | BY_22_M_03_O_0224_i01 | BC_WC | BC_WC | WC | WC | F2 | F2 | BC_WC | BC_WC | wildcat | WC |
|  | BY_22_M_03_O_0254_i01 | BC_WC | BC_WC | BC_WC | BC_WC | F2 | F2 | WC | WC | wildcat | WC |
|  | HE_00_F_03_O_0699_i01 | F2 | BC_DC | F1 | BC_DC | BC_DC | F1 | F2 | F1 | wildcat | F1 |
|  | HE_03_F_18_B_0737_i05^X)^ | BC_WC | F1 | F1 | F1 | F1 | BC_WC | BC_WC | BC_WC | wildcat | BC_WC |
|  | HE_03_M_03_O_0791_i01^X)^ | F1 | F1 | F1 | F1 | BC_WC | BC_WC | F2 | BC_WC | wildcat | F1 |
|  | HE_22_M_15_O_1201_i05^X)^ | F2 | F2 | F2 | F2 | BC_WC | BC_WC | F1 | F1 | wildcat | bad quality |
|  | HE_23_M_09_O_1100_i03^X)^ | F2 | F2 | F2 | F2 | BC_DC | BC_DC | BC_WC | BC_WC | wildcat | F2 |
|  | HE_23_M_12_O_0824_i04 | BC_WC | BC_WC | WC | WC | F2 | F2 | BC_WC | BC_WC | wildcat | WC |
|  | HE_34_M_03_O_0514_i01^X)^ | F2 | F2 | DC | DC | DC | BC_DC | F2 | F2 | unassigned | DC |
|  | NI_00_M_12_O_0120_i04 | DC | DC | DC | DC | BC_DC | BC_DC | F1 | F1 | wildcat | F1 |
|  | NI_06_F_09_G_0185_i03 | BC_WC | BC_WC | F1 | BC_WC | F2 | F1 | BC_WC | F1 | wildcat | not assigned |
|  | NI_12_M_06_G_0459_i02 | BC_WC | BC_WC | BC_WC | BC_WC | F2 | F2 | F2 | F2 | domestic | F1 |
|  | RP_22_M_15_B_0054_i05^X)^ | F2 | BC_DC | F1 | BC_DC | BC_DC | F2 | F2 | F1 | wildcat | not run |
|  | ST_22_M_03_O_0124_i01 | BC_WC | BC_WC | BC_WC | BC_WC | F2 | F2 | F1 | F1 | wildcat | F1 |
|  | ST_22_M_03_O_0125_i01 | BC_WC | BC_WC | F1 | BC_WC | F2 | F2 | F2 | F1 | wildcat | F1 |
|  | TH_00_M_06_G_0157_i01 | F2 | F2 | F2 | F2 | BC_WC | BC_DC | BC_WC | BC_WC | wildcat | WC |
|  | TH_22_M_03_G_0241_i01 | BC_WC | BC_WC | WC | WC | F2 | WC | BC_WC | BC_WC | wildcat | WC |

^X)^ individuals that were run in both datasets (central and west)
